# Picornavirus 3D polymerase inhibits antiviral innate immunity by blocking the activation of JAK-STAT signaling pathway

**DOI:** 10.1128/mbio.01666-25

**Published:** 2025-09-11

**Authors:** Kangli Li, Xiangle Zhang, Chen Liu, Guoliang Zhu, Shuo Wang, Dandan Dong, Xiaodan Wen, Weijun Chao, Baohong Liu, Ruoqing Mao, Yi Ru, Hong Tian, Huanan Liu, Bo Yang, Jijun He, Jianhong Guo, Jianye Dai, Fan Yang, Zixiang Zhu, Haixue Zheng

**Affiliations:** 1 State Key Laboratory for Animal Disease Control and Prevention, College of Veterinary Medicine, Lanzhou University, Lanzhou Veterinary Research Institute Chinese Academy of Agricultural Sciences 111658 , Lanzhou, China; 2 WOAH/National reference laboratory for foot-and-mouth disease, Lanzhou, China; 3 Gansu Province Research Center for Basic Disciplines of Pathogen Biology 625735 , Lanzhou, China; 4 School of Pharmacy, Lanzhou University 12426, Lanzhou, China; Cornell University, Ithaca, New York, USA

**Keywords:** picornavirus, innate immune response, 3D protein, JAK1, ubiquitination, RNF125

## Abstract

Picornaviruses are known for their rapid replication rate, which is a key characteristic that allows them to quickly infect host cells and produce progeny viruses. The interactions with host cells established by the virus to bypass host defense are essential for their rapid replication. The 3D protein, identified as the RNA-dependent RNA polymerase (RdRp) of picornaviruses, is central to viral replication. In this study, we delineated a conserved strategy employed by picornavirus 3D proteins to counteract the antiviral response elicited by interferons. Senecavirus A (SVA) 3D protein targets and hijacks JAK1, a pivotal component of the innate immune cascade, thereby decreasing the expression of a spectrum of interferon-stimulated antiviral genes. The interaction between the N-terminal region of SVA 3D (amino acids 1–152) and the FERM domain of JAK1 is responsible for the suppression of JAK-STAT signaling. Mechanistically, SVA 3D recruits the E3 ubiquitin ligase RNF125, inducing the K48-linked polyubiquitination of JAK1 at K205 and K249, which ultimately leads to the proteasomal degradation of JAK1. 3D proteins from other picornaviruses (FMDV, EMCV, and EV71) also suppress JAK1-mediated antiviral response. Collectively, these findings elucidate the suppressive regulatory mechanisms of picornaviruses 3D, highlighting a prevalent immune evasion tactic among picornaviruses, which will provide insights for developing antiviral strategies against picornaviruses.

**IMPORTANCE:**

Picornaviruses can establish interactions with host cells to bypass host defense mechanisms. The highly conserved viral polymerase 3D protein of picornavirus broadly inhibited JAK-STAT signaling and promoted viral replication. Specifically, SVA 3D induces the K48-linked ubiquitination of JAK1 through recruitment of the E3 ubiquitin ligase RNF125. Similarly, FMDV, EMCV, and EV71 3D proteins act as negative regulators to inhibit JAK-STAT pathway activation. These findings unveil a common immune suppression strategy employed by picornaviruses, thereby advancing our understanding of picornavirus pathogenesis and opening avenues for developing antiviral strategies against picornaviruses.

## INTRODUCTION

Picornaviruses are among the most frequently encountered infectious agents in humans and animals, causing a spectrum of diseases including hand, foot, and mouth disease, gastrointestinal infections, respiratory tract infections, and central nervous system disorders (1–4). Picornaviruses are nonenveloped, positive-sense, single-stranded RNA viruses. The viral genome consists of a 5′ untranslated region (5′ UTR), a large open reading frame (ORF), and a 3′ UTR (5). The ORF encodes four structural proteins (VP4, VP2, VP3, and VP1) and seven non-structural proteins (2A, 2B, 2C, 3A, 3B, 3C, and 3D), which are crucial for protein processing and viral replication (6).

The innate immune system is the primary defense against viral infections. Upon viral infection, pattern recognition receptors (PRRs) of the innate immune system detect viral elements, triggering a signaling cascade that leads to the production of interferons (IFNs) and pro-inflammatory cytokines. The produced IFNs activate the JAK-STAT signaling pathway, inducing the expression of interferon-stimulated genes (ISGs), which are pivotal in disrupting viral protein synthesis and inhibiting viral replication. Picornaviruses are widely known for their rapid replication rate, which is a key characteristic that allows them to quickly infect host cells and produce progeny viruses. They have evolved sophisticated mechanisms to evade host innate immunity, which is critical for their rapid replication.

Senecavirus A (SVA) is particularly adept at suppressing the host innate immune response, with the viral 3C protein playing a significant role. The 3C protein cleaves STAT1, STAT2, IRF9, and degrades KPNA1, inhibiting the formation and nuclear import of ISGF3 complex and blocking the activation of the JAK-STAT signaling pathway (7, 8). Similarly, foot-and-mouth disease virus (FMDV) 3C protein blocks the nuclear translocation of STAT1/STAT2 to inhibit the activation of JAK-STAT signaling (9). FMDV VP3 degrades JAK1 to inhibit IFN-induced signal transduction (10). FMDV Lb protease directly cleaves STAT1 and STAT2 to antagonize IFN-induced signaling (11). EV71 infection blocks the nuclear transport of STAT1 through induction of the degradation of KPNA1 (12). Currently, the antagonistic effects of picornaviruses on the JAK-STAT pathway are primarily attributed to 3C, VP3, and L proteins, while the potential roles of other viral proteins in controlling host innate immunity remain unknown.

In this study, we identified the interaction between the aa1–152 region of the N-terminal domain of SVA 3D and JAK1, which mediates the degradation of JAK1 and subsequently inhibits the activation of the JAK-STAT signaling pathway. The presence of 3D enhances the K48-linked polyubiquitination of JAK1 at the residues K205 and K249, with the involvement of the E3 ubiquitin ligase RNF125 in this process. In addition, we observed that 3D proteins from other picornaviruses, including FMDV, EMCV, and EV71, broadly inhibited JAK-STAT signaling and promoted viral replication. These results suggest that picornavirus 3D protein not only facilitates viral replication by functioning as RNA-dependent RNA polymerases (RdRp) but also serves as an antagonist of innate immunity to inhibit the JAK-STAT signaling pathway, thereby promoting viral replication.

## RESULTS

### IFN-β inhibited SVA replication, and SVA impaired IFN-β-induced transcription of antiviral genes

**Fig 1:**
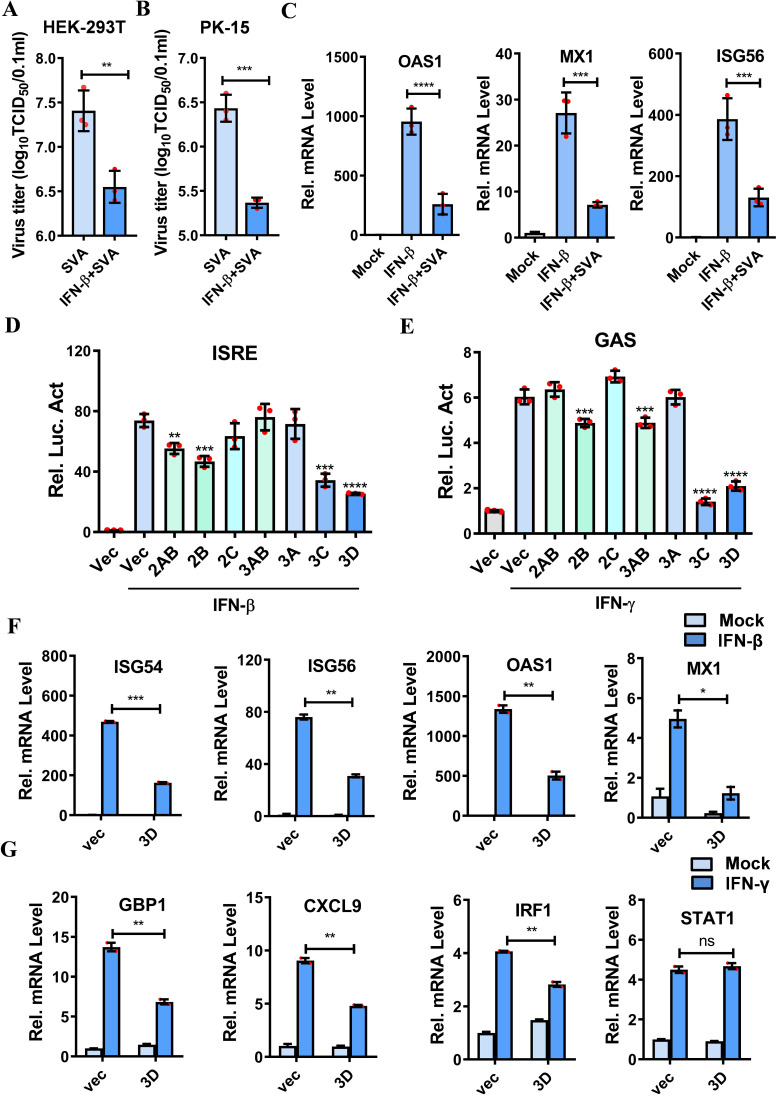
SVA 3D protein inhibited IFN signaling pathway. (**A, B**) HEK-293T cells (**A**) and PK-15 cells (**B**) were pretreated with human IFN-β (1,000 U/mL) or porcine IFN-β (1000 U/mL) for 2 h, respectively, followed by infection with GFP-SVA (MOI = 0.5). At 12 h post-infection (hpi), the supernatants were harvested for determining viral titers. (**C**) The expression of *OAS1*, *MX1,* and *ISG56* was detected by qPCR in HEK-293T cells pretreated with human IFN-β followed by SVA infection or mock infection. (**D, E**) HEK-293T cells were co-transfected with *ISRE* (50 ng) or *GAS* (100 ng) luciferase reporter plasmids, along with an internal control plasmid PRL-TK (5 ng) and either an empty vector (Vec) or the indicated viral protein-expressing plasmids (50 ng) for 24 h, and the cells were treated with IFN-β (1,000 U/mL) to activate the *ISRE* promoter (**D**) or with IFN-γ (1,000 U/mL) to activate the *GAS* promoter (**E**) for an additional 12 h. The luciferase activity was measured by dual luciferase assay. (**F**) The mRNA levels of *ISG54*, *ISG56*, *OAS1,* and *MX1* in HEK-293T cells transfected with either Vec or Flag-3D plasmids, followed by treatment with IFN-β or solvent control (Mock), were measured by qPCR. (**G**) The mRNA levels of *GBP1*, *CXCL9*, *IRF1,* and *STAT1* in HEK-293T cells transfected with either Vec or Flag-3D plasmids, followed by treatment with IFN-γ or solvent control (Mock), were measured by qPCR. All experiments were repeated three times, with similar results. *P < 0.05, **P < 0.01, ***P < 0.001, ****P < 0.0001, ns, not significant.

IFN-β, as an important member of type I IFNs, plays a central role in antiviral immunity. It significantly inhibits the replication of picornaviruses by activating the downstream JAK-STAT signaling pathway (9, 11, 12). To assess the antiviral impact of IFN-β on SVA replication, HEK-293T and PK-15 cells were treated with human and porcine IFN-β for 2 h, respectively, prior to infection with GFP-SVA at a multiplicity of infection (MOI) of 0.5. The GFP fluorescence was monitored under a fluorescence microscope. At 12 h post-infection (hpi), a notable decrease in GFP fluorescence was observed in IFN-β-pretreated cells compared to the solvent controls. The replication of SVA was characterized by detecting the 3D gene transcript in both cell lines using RT-PCR (Fig. S1A and B) and assessing the expression of VP2 and 3D proteins by western blotting (Fig. S1C and D). It revealed a significant suppression of SVA replication at both the protein and mRNA levels. In addition, the cell culture supernatants were collected for measuring the viral titers. The results indicated that IFN-β treatment significantly reduced viral titers in both HEK-293T (Fig. 1A) and PK-15 (Fig. 1B) cells compared to solvent control cells. These data indicate that IFN-β exerts a significant inhibitory effect on SVA replication in both human and porcine cells.

Activation of the type I IFN signaling pathway induces the expression of hundreds of ISGs. To investigate the effect of SVA infection on the IFN-β-mediated signaling cascade, we conducted an analysis of ISG expression in IFN-β-treated cells followed by SVA infection or mock infection. The results showed that the transcriptional levels of *OAS1*, *MX1,* and *ISG56* in HEK-293T cells were highly increased upon IFN-β treatment. However, this upregulation was markedly inhibited by SVA infection (Fig. 1C). These observations indicate that the IFN-β signaling is significantly impaired during SVA infection.

### SVA 3D protein inhibited IFN-mediated signaling and decreased ISG expression

IFNs are crucial components of the innate immune system. We determined that SVA infection suppresses the expression of ISGs triggered by IFN-β, raising the question of which viral proteins are responsible for this inhibition. To identify the SVA proteins that counteract innate immunity, we evaluated the effects of SVA viral protein on the activation of IFN-β-induced *ISRE* promoter and IFN-γ-induced *GAS* promoter. Our screening revealed that SVA 3D protein notably inhibits the activation of both the *ISRE* and *GAS* promoters (Fig. 1D and E). The successful expression of various SVA proteins was verified by western blotting (Fig. S1E and F). The influence of the 3D protein on the expression of ISGs induced by IFN-β and IFN-γ was further investigated. We observed that the mRNA expression of *ISG54*, *ISG56*, *OAS1,* and *MX1*, which are stimulated by IFN-β, was significantly reduced in HEK-293T cells in the presence of 3D protein (Fig. 1F). Similarly, the mRNA levels of *GBP1*, *CXCL9,* and *IRF1*, induced by IFN-γ, were downregulated in the presence of 3D, with STAT1 not exhibiting a significant change (Fig. 1G). In addition, the IFN-β-induced upregulation of mRNA levels of *ISG15*, *ISG54*, *OAS1,* and *MX1* in PK-15 cells was significantly repressed by 3D (Fig. S1G). These results indicate that SVA 3D protein plays a crucial role in inhibiting IFN-mediated signaling and the expression of ISGs.

### SVA 3D inhibited the expression of JAK1

**Fig 2:**
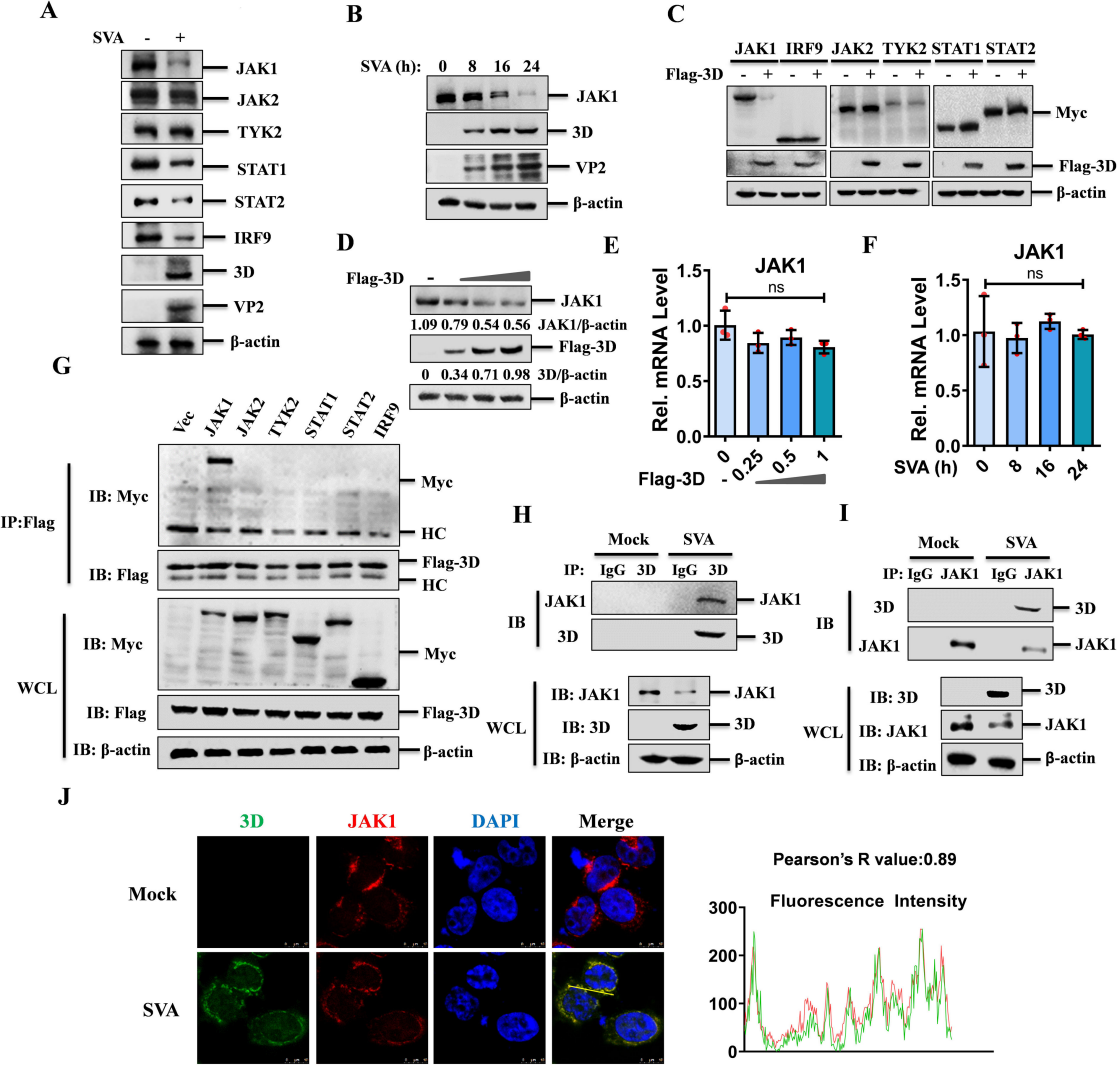
SVA 3D protein inhibited the expression of JAK1 and interacted with it. (**A**) HEK-293T cells were mock-infected or infected with SVA (MOI = 0.5) for 12 h, and the cell lysates were analyzed by western blotting using the specified antibodies. (**B**) HEK-293T cells were infected with SVA (MOI = 0.5) for 0, 8, 16, and 24 h, respectively, and then analyzed by western blotting using the specified antibodies. (**C**) HEK-293T cells were co-transfected with either an empty vector or plasmids expressing Flag-tagged 3D, together with plasmids encoding Myc-tagged innate immune molecules (JAK1, JAK2, TYK2, STAT1, STAT2, or IRF9). The cells were lysed at 24 hpt and analyzed by western blotting using the indicated antibodies. (**D, E**) HEK-293T cells were transfected with 0, 0.25, 0.5, or 1 µg of Flag-3D expressing plasmids for 24 h. The expression levels of endogenous JAK1 protein were assessed by western blotting (**D**), and the JAK1 mRNA levels were quantified by qPCR (**E**). (**F**) HEK-293T cells were infected with SVA (MOI = 0.5) for 0, 8, 16, and 24 h, respectively. Total RNA was extracted, and the mRNA expression levels of JAK1 were analyzed by qPCR. (**G**) HEK-293T cells were co-transfected with Flag-3D along with empty vector (Vec) or various Myc-tagged innate immune molecule-expressing plasmids (JAK1, JAK2, TYK2, STAT1, STAT2, or IRF9). At 36 hpt, the cell lysates were subjected to Co-IP assay analysis. The immunoprecipitated proteins and whole-cell lysates (WCL) were analyzed by western blotting using the specified antibodies. (**H, I**) HEK-293T cells were mock-infected or infected with SVA at an MOI of 0.5 for 12 h. Cell lysates were then immunoprecipitated with anti-3D (**H**) or anti-JAK1 (**I**) antibodies. (**J**) HEK-293T cells were mock-infected or infected with SVA (MOI = 0.1) for 10 h, after which the colocalization of JAK1 (red) and 3D (green) was assessed by immunofluorescence assay (IFA). Nuclei were counterstained with DAPI (blue). HC: heavy chain.

To investigate whether 3D protein exerts a negative regulatory effect on IFN-mediated signaling through modulation of the JAK-STAT pathway components, we examined the expression levels of JAK1, JAK2, TYK2, STAT1, STAT2, and IRF9 in both mock-infected and SVA-infected HEK-293T cells. The results revealed that SVA infection led to a significant downregulation of JAK1, STAT1, STAT2, and IRF9 expression (Fig. 2A). To further validate this finding, the temporal expression profiles of JAK1, STAT1, STAT2, and IRF9 at 0, 8, 16, and 24 hpi by SVA were detected. As the SVA infection progressed, the protein levels of JAK1, STAT1, STAT2, and IRF9 exhibited a consistent and progressive decrease (Fig. 2B and Fig. S2A). SVA 3C protein degrades STAT1, STAT2, and IRF9 (8), and this is consistent with the downregulation of STAT1, STAT2, and IRF9 by SVA infection. To identify the key molecules in the JAK-STAT pathway targeted by 3D protein, HEK-293T cells were transfected with either an empty vector or a plasmid expressing Flag-tagged 3D protein, together with Myc-tagged plasmids encoding various components of the innate immune response (JAK1, JAK2, TYK2, STAT1, STAT2, or IRF9). Western blot analysis showed that 3D specifically and significantly downregulated the expression of JAK1, while having no significant effect on the expression of the other components (Fig. 2C). Furthermore, a dose-dependent assay was performed, which revealed that the overexpression of SVA 3D reduced JAK1 expression in a dose-dependent manner in both HEK-293T cells (Fig. S2B) and PK-15 cells (Fig. S2C), but the expression of STAT1 (Fig. S2D) and STAT2 (Fig. S2E) was not affected by 3D overexpression. To verify the degradation of JAK1 mediated by 3D, we transfected cells with plasmids expressing Flag-tagged 3D. A dose-dependent reduction in endogenous JAK1 expression was consistently observed in both Flag-3D expressing HEK-293T cells (Fig. 2D) and PK-15 cells (Fig. S2F). In addition, our results indicated that SVA 3D had no significant effect on JAK1 mRNA expression in either HEK-293T cells (Fig. 2E) or PK-15 cells (Fig. S2G). To investigate the impact of SVA infection on JAK1 transcription, HEK-293T cells and PK-15 cells were infected with SVA for 0, 8, 16, and 24 h. We found that the mRNA expression of JAK1 remained unchanged as the infection progressed in both HEK-293T cells (Fig. 2F) and PK-15 cells (Fig. S2H). These findings suggest that SVA 3D specifically degrades JAK1 at the protein level, thereby inhibiting IFN-mediated signaling, without significantly affecting JAK1 mRNA expression.

### SVA 3D protein interacted with the tyrosine kinase JAK1

To determine whether 3D inhibits the activation of the JAK-STAT pathway by interacting with its components, we assessed the interaction between SVA 3D and the key proteins JAK1, JAK2, TYK2, STAT1, STAT2, and IRF9 using co-immunoprecipitation (Co-IP) assays. The results indicated that Flag-3D specifically interacted with Myc-JAK1 (Fig. 2G). This interaction was further confirmed by a reverse immunoprecipitation assay, which showed that only Myc-JAK1 was able to pull down Flag-3D (Fig. S2I). To confirm the interaction between 3D and JAK1, HEK-293T cells were transfected with Flag-3D and Myc-JAK1-expressing plasmids, the cell lysates were used for Co-IP experiments using both anti-Flag antibodies and IgG control, which validated that Flag-3D specifically interacted with Myc-JAK1 (Fig. S2J). Similarly, the subsequent reverse Co-IP experiments showed consistency of interaction (Fig. S2K). In addition, we investigated the subcellular localization of SVA 3D and JAK1. HEK-293T cells were transfected with Myc-JAK1-expressing plasmids, and the cells were subjected to SVA infection or mock infection. A colocalization of JAK1 and 3D was clearly observed in the cytoplasm after SVA infection (Fig. S2L).

To extend our findings to a viral infection context, HEK-293T cells were mock-infected or infected with SVA for 12 h. Cell lysates were then immunoprecipitated with anti-3D antibodies and subjected to western blotting analysis, which demonstrated the association of 3D with JAK1 during SVA infection (Fig. 2H). A reciprocal immunoprecipitation using anti-JAK1 antibodies also showed that JAK1 could precipitate SVA 3D (Fig. 2I). Furthermore, the co-localization of 3D with intracellular JAK1 upon SVA infection was examined, which revealed that 3D co-localized with JAK1, and SVA infection diminished JAK1 fluorescence (Fig. 2J), more cells are shown in Fig. S2M. Taken together, these results confirmed that SVA 3D interacts with host JAK1 protein during viral infection.

### SVA 3D protein blocked phosphorylation and dimerization of STATs

**Fig 3:**
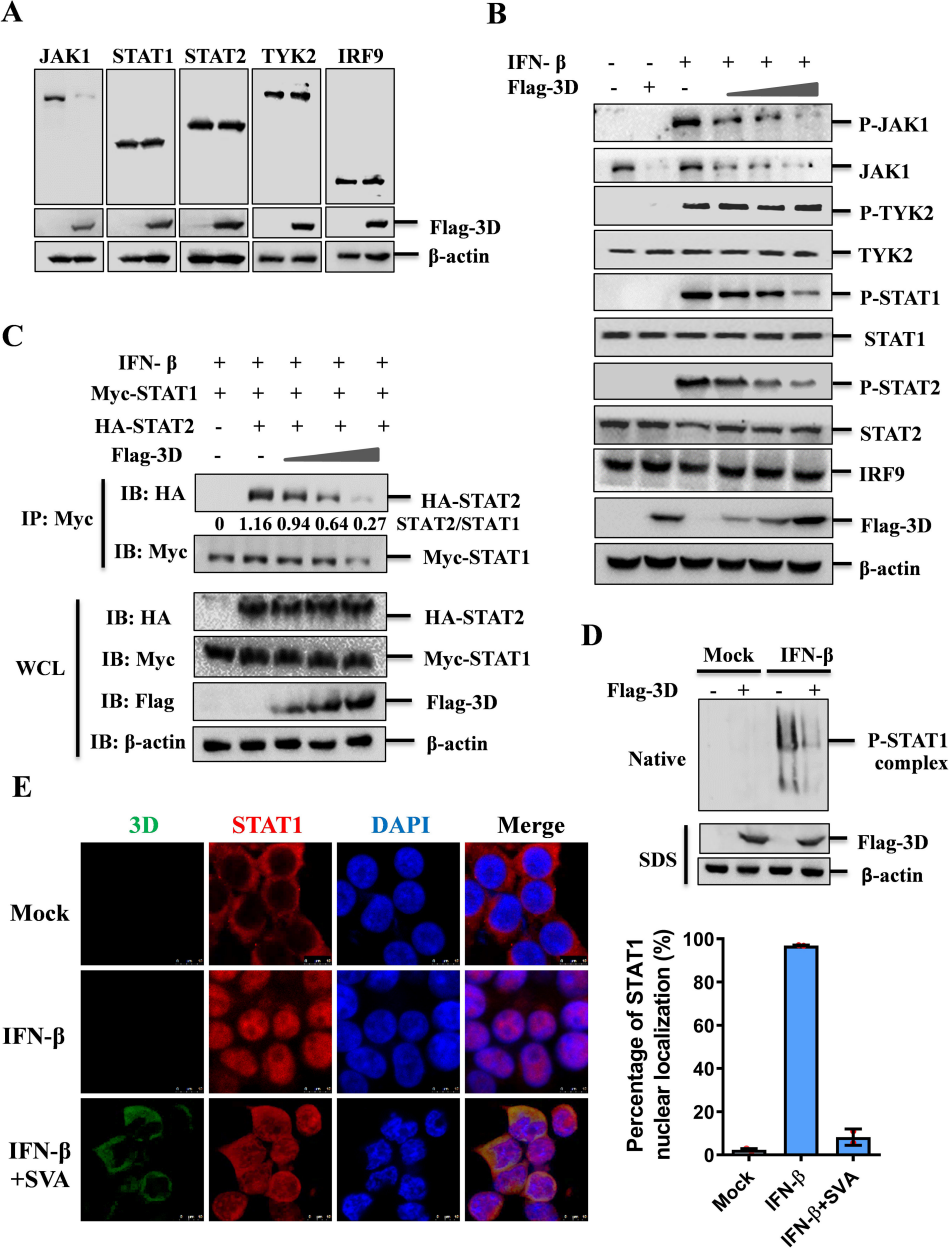
SVA 3D protein suppressed phosphorylation and dimerization of STATs. (**A**) HEK-293T cells were transfected with either an empty vector or Flag-3D expressing plasmids. At 24 hpt, the cell lysates were analyzed by western blotting with the specified antibodies. (**B**) HEK-293T cells were transfected with increasing amounts of Flag-3D expressing plasmids (0, 0.25, 0.5, or 1 µg). At 24 hpt, the cells were treated with IFN-β (1,000 U/mL) or solvent control for 30 min, after which the cell lysates were analyzed by western blotting with the indicated antibodies. (**C**) HEK-293T cells were co-transfected with Myc-STAT1, HA-STAT2, or HA-vector, and increasing amounts Flag-3D-expressing plasmids. At 36 hpt, the cells were treated with IFN-β (1,000 U/mL) for 30 min. The cells were lysed and immunoprecipitated with anti-Myc antibodies. The immunoprecipitated proteins and WCL were analyzed by western blotting using the specified antibodies. (**D**) HEK-293T cells were transfected with empty vector or Flag-3D-expressing plasmids. At 24 hpt, the cells were treated with IFN-β (1,000 U/mL) or solvent control for 30 min. The cell lysates were then analyzed by native PAGE and SDS-PAGE using the specified antibodies. (**E**) HEK-293T cells mock-infected or infected with SVA (MOI = 0.1) for 10 h, followed by IFN-β (1,000 U/mL) or mock treatment for 30 min. The subcellular localization of STAT1 and SVA-3D was assessed by IFA. Nuclei were stained with DAPI (blue), and fluorescence was visualized for 3D (green) and STAT1 (red).

To validate the influence of 3D on the JAK-STAT signaling pathway, we assessed the impact of 3D on the endogenous levels of JAK1, STAT1, STAT2, TYK2, and IRF9. The results indicated that 3D selectively targeted JAK1 for degradation (Fig. 3A). Furthermore, in the presence of 3D protein, not only was IFN-β-induced JAK1 phosphorylation and endogenous JAK1 expression significantly inhibited, but also was the phosphorylation of STAT1 and STAT2 suppressed, with other proteins remaining unaffected (Fig. 3B). We further explored the impact of 3D protein on the formation of the STAT1-STAT2 complex in response to IFN-β and found that increased 3D levels corresponded to a reduced amount of STAT2 precipitated by STAT1 (Fig. 3C). We also explored the influence of 3D on STATs dimerization and observed that, while IFN-β stimulation induced STAT1 phosphorylation and dimerization, the presence of 3D impaired this effect, suggesting the inhibitive effect of 3D on JAK-STAT pathway signal transduction (Fig. 3D). To confirm the suppressive effect of SVA infection on STAT1 nuclear translocation, HEK-293T cells were infected with SVA followed by IFN-β treatment, and the subcellular localization of STAT1 was determined. Our observations revealed that STAT1 nuclear translocation was initiated by IFN-β treatment, but this process was inhibited by SVA infection (Fig. 3E). More cells at low magnification are shown in Fig. S3. These data indicate that the degradation of JAK1 by 3D hinders the phosphorylation and dimerization of STATs in SVA-infected cells, which are essential for JAK-STAT pathway signal transduction.

### SVA 3D promoted the degradation of JAK1 through the proteasome pathway

**Fig 4:**
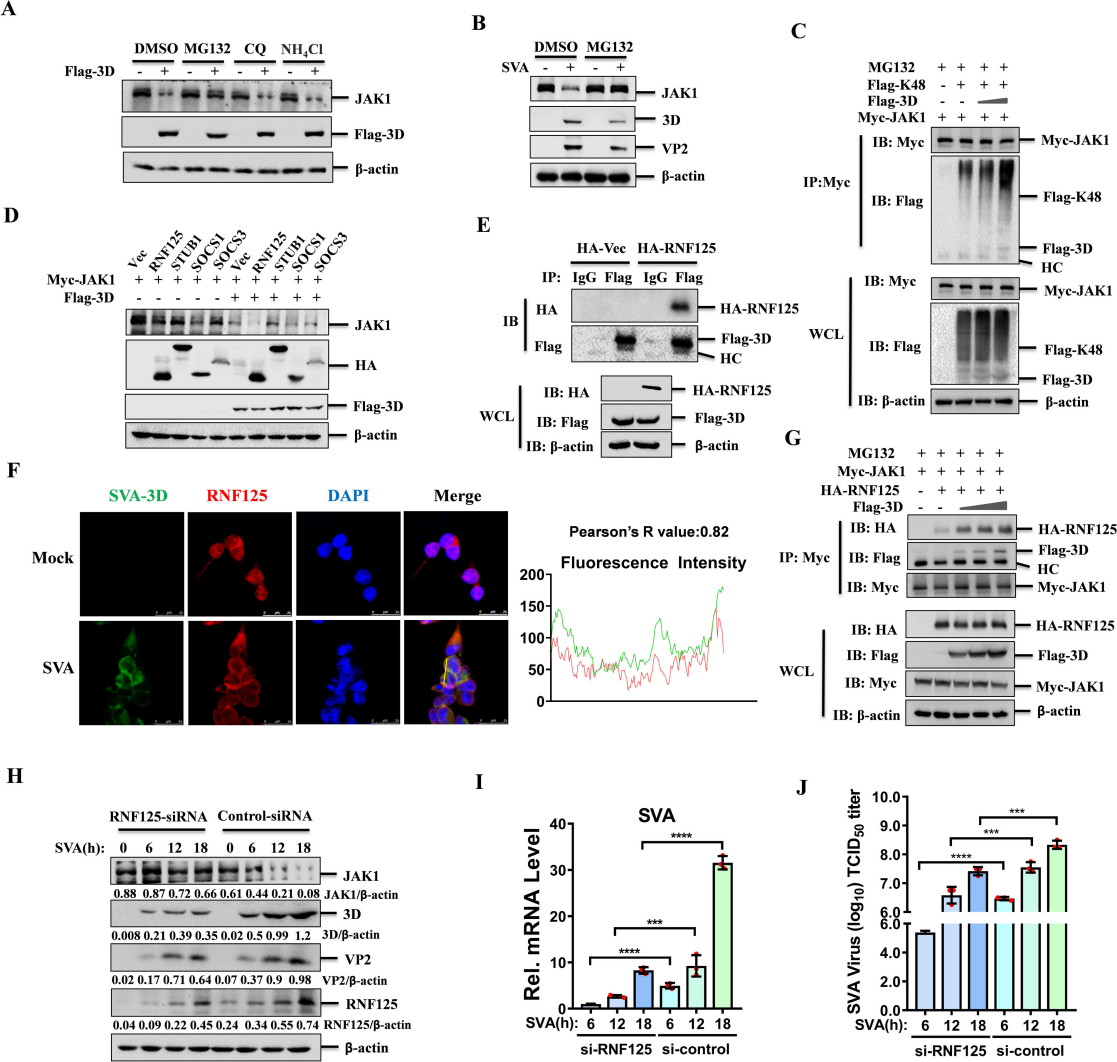
SVA 3D protein enhanced RNF125-mediated K48-linked ubiquitination and degradation of JAK1. (**A**) HEK-293T cells were co-transfected with empty vector or Flag-3D. At 24 hpt, the cells were treated with 20 µM MG132, 50 µM chloroquine (CQ), or 50 µM ammonium chloride (NH_4_Cl) for 6 h. The cell lysates were analyzed by western blotting with the indicated antibodies. (**B**) HEK-293T cells mock-infected or infected with SVA (MOI = 0.5) for 12 h, followed by MG132 (20 µM) or DMSO treatment for another 6 h. The cell lysates were then analyzed by western blotting using the indicated antibodies. (**C**) HEK-293T cells were co-transfected with Myc-JAK1, Flag-K48, or Flag-vector, and increasing amounts of Flag-3D expressing plasmids, followed by treatment with MG132. The immunoprecipitated proteins and WCL were subjected to western blotting analysis using the indicated antibodies. (**D**) HEK-293T cells were transfected with HA-tagged E3 ubiquitin ligases (RNF125, STUB1, SOCS1, or SOCS3), and Flag-vector or Flag-3D for 24 h. The cell lysates were analyzed by western blotting with the indicated antibodies. (**E**) HEK-293T cells were transfected with Flag-3D and HA-RNF125. At 36 hpt, the cell lysates were immunoprecipitated with anti-Flag antibodies or IgG, and then analyzed by western blotting using the indicated antibodies. (**F**) Colocalization of RNF125 (red) and 3D (green) in SVA-infected HEK-293T cells was visualized through IFA. Nuclei were stained with DAPI (blue). (**G**) HEK-293T cells were co-transfected with Myc-JAK1, HA-RNF125, or HA-Vector, and increasing amounts of Flag-3D expressing plasmids in the presence of MG132. The cell lysates were subjected to co-immunoprecipitation and western blotting analyses using the indicated antibodies. (**H-J**) HEK-293T cells were transfected with Control siRNA or RNF125 siRNA (1 µg/mL) for 36 h, and then infected with SVA (MOI = 0.5) for 0, 6, 12, or 18 h. Cell lysates were analyzed by western blotting (**H**), the mRNA expression of SVA 3D was analyzed by qPCR (**I**), and the cell supernatants were harvested for determining viral titers (**J**).

The ubiquitin-proteasome and autophagy-lysosome systems are the primary mechanisms for protein degradation in eukaryotic cells. In our investigation into the degradation mechanism of JAK1 by 3D, we transfected HEK-293T cells with plasmids expressing Flag-3D and treated them with either proteasome inhibitor MG132 or lysosome inhibitors chloroquine (CQ) and ammonium chloride (NH_4_Cl). The results showed that MG132 treatment could reverse the 3D-induced degradation of JAK1, whereas CQ or NH_4_Cl had no such effect (Fig. 4A). A dose-response experiment with MG132 revealed that increasing concentrations of MG132 progressively enhanced the recovery of JAK1 levels (Fig. S4A). We also examined the effect of MG132 on the expression of JAK1 during SVA infection, and the results showed that the treatment of MG132 could restore the downregulation of JAK1 (Fig. 4B). Thus, it suggested that 3D mediates JAK1 degradation via the proteasome pathway.

Given the significance of ubiquitination in proteasomal degradation, we examined whether 3D degrades JAK1 by promoting ubiquitination of JAK1. Consistent with our hypothesis, we observed that 3D could enhance the ubiquitination of JAK1 in a dose-dependent manner (Fig. S4B). Ubiquitination involves various linkages, including K6-, K11-, K27-, K29-, K33-, K48-, and K63-linkage. To identify the specific ubiquitination manner involved in 3D-mediated JAK1 degradation, we performed Co-IP experiments after transfecting HEK-293T cells with plasmids expressing Myc-JAK1, Flag-3D, and Flag-tagged mutant Ub (K6-, K29-, K48-, K63-, K11-, K33-, or K27-only). We found that the presence of 3D increased the amount of K48-linked ubiquitin pulled down by Myc-JAK1, while other ubiquitination mutants had no significant impact (Fig. S4B). In addition, 3D could enhance K48-linked ubiquitination of JAK1 in a dose-dependent manner (Fig. 4C). Consequently, these data suggested that 3D promotes K48-linked ubiquitination of JAK1.

### 3D enhanced the K48-linked ubiquitination and subsequent degradation of JAK1 by RNF125

3D induces the degradation of JAK1 through the proteasome pathway, despite lacking hydrolytic enzyme activity itself. This led us to hypothesize that 3D may function as a scaffold to bridge JAK1 with an E3 ligase, facilitating its ubiquitination and subsequent degradation. Previous studies have pinpointed several E3 ubiquitin ligases involved in JAK1 ubiquitination, such as RNF125, STUB1, SOCS1, and SOCS3 (13–16). To determine which E3 ligase is involved in 3D-mediated JAK1 degradation, we co-transfected HEK-293T cells with 3D and each of these E3 ligases. We observed that RNF125 overexpression downregulated JAK1 levels, and 3D could significantly enhance this degradation (Fig. 4D). Co-transfection of Flag-3D and HA-RNF125 revealed that 3D could immunoprecipitate RNF125 (Fig. 4E), and reciprocal immunoprecipitation confirmed the interaction between RNF125 and 3D (Fig. S4D). The subcellular localization analysis showed that 3D and RNF125 co-localized in the cytoplasm during SVA infection (Fig. 4F), and more cells are shown in Fig. S4E. Furthermore, overexpression of RNF125 was found to promote K48-linked ubiquitination of JAK1 (Fig. S4F). Based on these observations, we proposed that RNF125 is the E3 ubiquitin ligase responsible for mediating 3D-induced JAK1 degradation. This hypothesis was supported by further co-transfection experiments showing that 3D enhanced the interaction between JAK1 and RNF125 in a dose-dependent manner (Fig. 4G). To confirm the role of RNF125 in 3D-mediated JAK1 degradation, we used RNA interference to target RNF125 (Fig. S4G) and observed that JAK1 degradation was reversed in RNF125-knockdown cells (Fig. S4H). Moreover, RNF125 knockdown resulted in upregulated JAK1 expression and reduced viral replication (Fig. 4H and Fig. S4I). The replication of SVA was also inhibited in RNF125-knockdown cells at the mRNA level (Fig. 4I). In addition, cell culture supernatants were collected to measure viral titers, and the results showed that RNF125 knockdown significantly reduced viral titers (Fig. 4J). These results indicated the importance of this pathway in the host antiviral response. Taken together, these data suggest that 3D degrades JAK1 by recruiting the E3 ubiquitin ligase RNF125, thereby increasing K48-linked ubiquitination of JAK1 and impairing host antiviral response.

### The degradation of JAK1 by 3D was dependent on the K249 residue in JAK1 and the N-terminal region (aa1–152) of 3D

**Fig 5:**
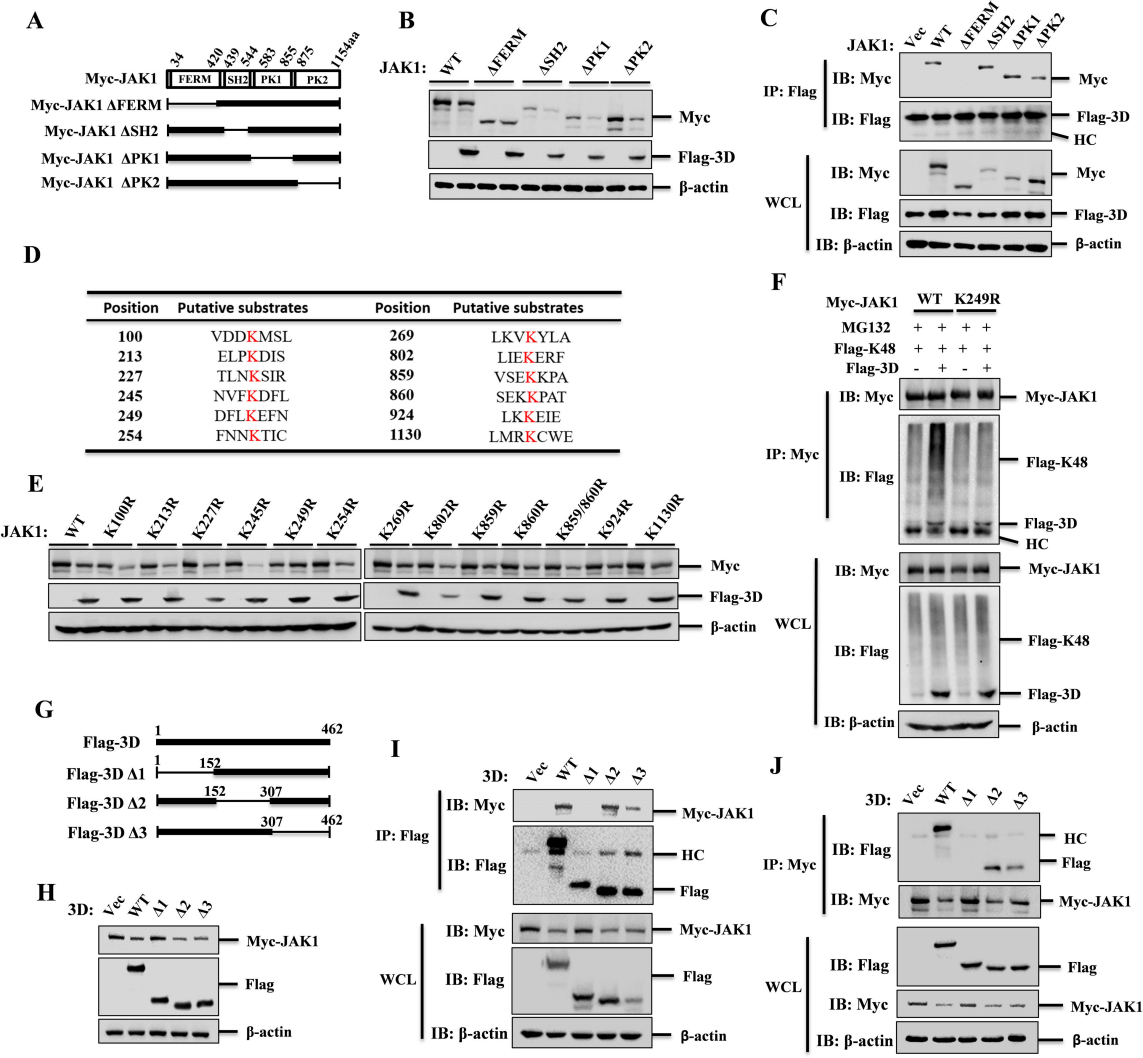
The K249 residues of JAK1 and the N-terminal aa1–152 region of 3D were essential for their function. (**A**) Schematic representation of Myc-tagged truncated JAK1 mutants. (**B**) HEK-293T cells were co-transfected with 1 µg of Myc-JAK1 or the indicated Myc-JAK1 mutants along with Flag-3D or an empty vector for 24 h. Expression of Myc-JAK1 and JAK1 mutants was detected by western blotting. (**C**) Co-IP analysis to assess the interaction between Flag-3D and Myc-JAK1 or the indicated JAK1 mutants. (**D**) Prediction of ubiquitination modification sites on JAK1 using CPLM 1.0 software. (**E**) HEK-293T cells were co-transfected with 1 µg of Myc-JAK1 or JAK1 lysine mutants along with Flag-3D or an empty vector for 24 h. Expression of Myc-JAK1 and the JAK1 lysine mutants was detected by western blotting. (**F**) Analysis of K48-linked ubiquitination of JAK1 and the JAK1-K249R mutant in HEK-293T cells co-transfected with Myc-JAK1 or Myc-JAK1-K249R, Flag-K48, and Flag-3D expressing plasmids in the presence of MG132. Cell lysates were immunoprecipitated with an anti-Myc antibody and subjected to western blotting analysis. (**G**) Schematic representation of a series of Flag-tagged truncated 3D mutants. (**H**) Detection of Myc-JAK1 expression in HEK-293T cells co-transfected with Flag-3D or Flag-3D mutants and Myc-JAK1 by western blotting. (**I, J**) HEK-293T cells were transfected with Myc-JAK1 and empty vector, Flag-3D or Flag-3D mutants expressing plasmids, cell lysates were subjected to western blotting analysis at 36 hpt with anti-Flag antibody (**I**). Reverse Co-IP was performed and analyzed using Myc antibodies (**J**).

JAK1 protein consists of four domains: a predicted FERM domain (aa34–420), a potential SH2 domain (aa439–544), a pseudokinase domain (PK1, aa583–855), and a canonical tyrosine kinase domain (PK2, aa875–1,154). To identify the critical regions within JAK1 targeted by 3D, we generated deletion mutants based on these domains (Fig. 5A). Our results revealed that deletion of the FERM domain abolished the ability of 3D to degrade JAK1, whereas deletion of the SH2, PK1, or PK2 domains in 3D did not affect this degradation (Fig. 5B). Further pull-down assay confirmed that the FERM domain was the primary binding region for 3D, not the SH2, PK1, or PK2 domains (Fig. 5C). 3D induces JAK1 degradation through promoting its K48-linked ubiquitination, leading us to investigate which lysine residues in JAK1 are targeted by 3D. The database of CPLM 1.0 was used to predict the ubiquitination sites within JAK1 (Fig. 5D). Mutational analysis showed that mutation of the lysine 249 to arginine (K249R) within the FERM domain blocked 3D-mediated JAK1 degradation, but the other mutations (K100R, K213R, K227R, K245R, K254R, K269R, K802R, K859R, K860R, K859/860R, K924R, or K1130R) did not affect the degradation of JAK1 by 3D (Fig. 5E). In addition, we assessed the impact of 3D on the K48 ubiquitination of the JAK1(K249R) mutant and found that while 3D could still interact with JAK1(K249R), it failed to promote its K48 ubiquitination, highlighting the importance of K249 for 3D-mediated ubiquitination of JAK1 (Fig. 5F). These data suggested that 3D interacts with the FERM domain of JAK1, utilizing the K249 residue to facilitate K48 ubiquitination and subsequent degradation of JAK1, thereby suppressing the antiviral effects mediated by IFN-β.

To identify the essential functional domains of SVA 3D in suppressing JAK1 expression, we constructed truncated mutants of 3D: Flag-3D-Δ1 (lacking aa1–152), Flag-3D-Δ2 (lacking aa153–307), and Flag-3D-Δ3 (lacking aa308–462) (Fig. 5G). Overexpression of Flag-3D-Δ1 did not lead to JAK1 degradation, whereas overexpression of Flag-3D, Flag-3D-Δ2, and Flag-3D-Δ3 significantly induced JAK1 degradation (Fig. 5H). Co-IP experiments revealed that Flag-3D-Δ1 completely disrupted the interaction between 3D and JAK1, while other 3D mutants retained the ability to interact with JAK1 (Fig. 5I and J). Thus, the N-terminal aa1–152 region of 3D is crucial for its ability to degrade JAK1.

### Predicting and verifying the key interaction residues between JAK1 and 3D

**Fig 6:**
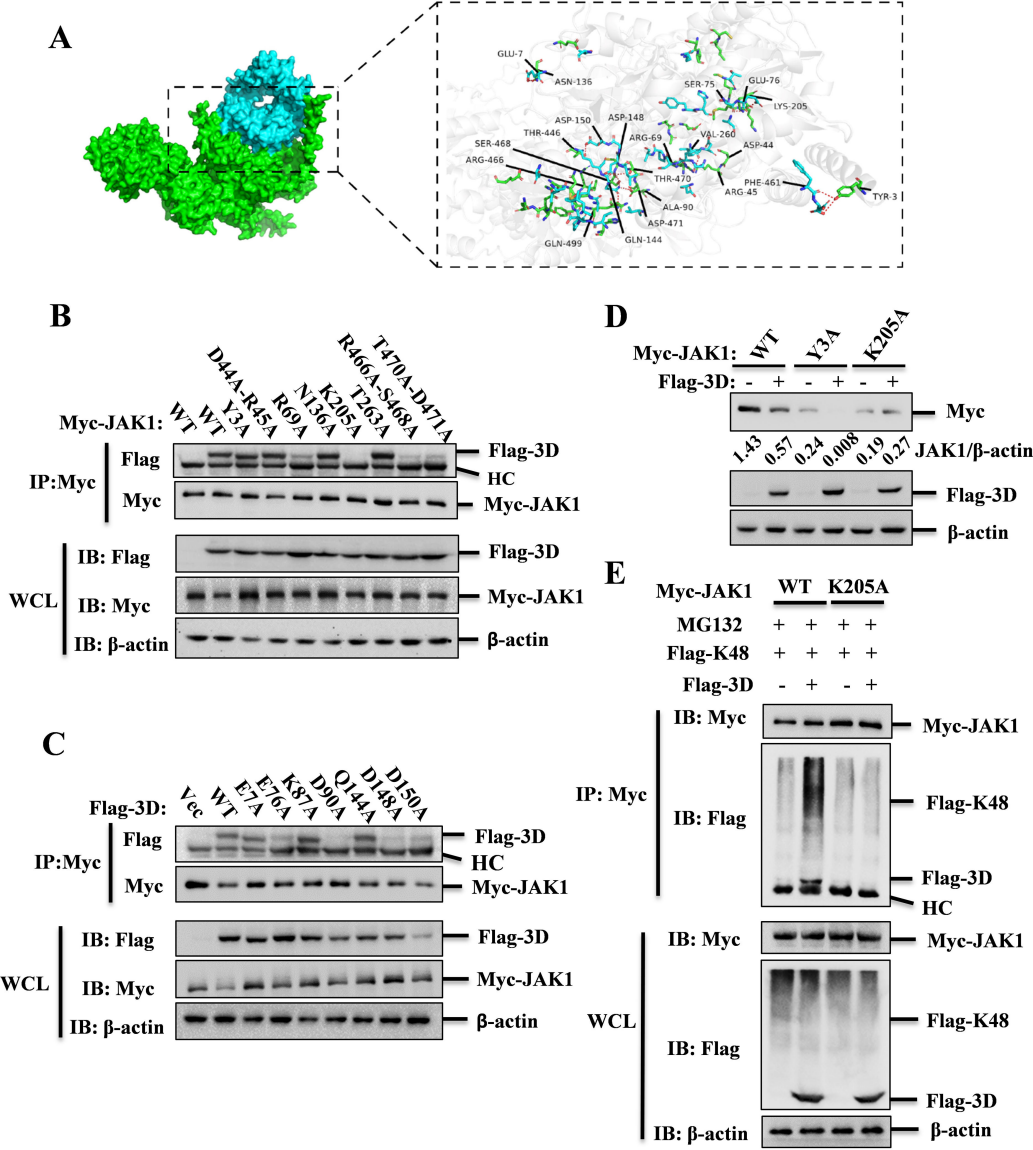
Prediction and verification key interaction residues between JAK1 and SVA-3D. (**A**) The interaction model between JAK1 and SVA-3D was constructed using the ZDOCK online service; the predicted interacting amino acids between JAK1 and 3D were highlighted using PyMOL software. (**B**) The interaction between 3D and JAK1 mutants was assessed in HEK-293T cells by co-transfecting Myc-tagged JAK1 or its mutants (Y3A, D44A-R45A, R69A, N136A, K205A, T263A, T466A-S468A, or T470A-D471A) with Flag-tagged 3D plasmids. Cell lysates were immunoprecipitated with anti-Myc antibodies at 36 hpt and subjected to western blotting analysis. (**C**) Co-IP assays were performed using anti-Myc antibodies in HEK-293T cells to detect the interaction of JAK1 and Flag-3D or Flag-3D mutants (E7A, E76A, K87A, D90A, Q144A, D148A, and D150A). (**D**) HEK-293T cells were transfected with empty vector or Flag-3D and Myc-JAK1 or its mutants expressing plasmids for 24 h. Expression of Myc-JAK1 and the indicated mutants were detected by western blotting. (**E**) Analysis of K48-linked ubiquitination of JAK1 or JAK1-K205A mutant in the absence or presence of SVA-3D by Co-IP assays.

The K249 residue of JAK1 was found to be non-essential for the interaction between JAK1 and 3D. To determine the interaction interfaces and residues mediating the JAK1-3D interaction, we utilized SWISS-MODEL online services for homologous modeling to construct models of the 3D and JAK1 proteins. Subsequently, we employed the ZDOCK online service (https://zdock.wenglab.org/) to predict the interactions between 3D and JAK1. The predictions suggested that JAK1 forms two salt bridges, four attractive charge interactions, and eight hydrogen bonds with 3D. Specifically, R69 and K205 of JAK1 form salt bridges with D90 and E76 of 3D, respectively. In addition, R45, R466, R466, and D44 of JAK1 engage in attractive charge interactions with D261, D148, D150, and K87 of 3D. Y3, Y3, S468, T470, S468, D471, T263, and N136 of JAK1 form hydrogen bonds with F461, D462, D148, D148, Q144, Q144, H256, and E7 of 3D, respectively (Fig. 6A and Table 1).

**TABLE 1:** The crucial residues involved in JAK1 and 3D interaction

JAK1	3D	Interaction type
R69	D90	Salt bridge
K205	E76	Salt bridge
R45	D261	Attractive charge
R466	D148	Attractive charge
R466	D150	Attractive charge
D44	K87	Attractive charge
Y3	F461	Hydrogen bond
Y3	D462	Hydrogen bond
S468	D148	Hydrogen bond
T470	D148	Hydrogen bond
S468	Q144	Hydrogen bond
D471	Q144	Hydrogen bond
T263	H256	Hydrogen bond
N136	E7	Hydrogen bond

To eliminate the potential influence of truncations on the overall structures and to ensure the accuracy of identifying truncations in our previous experiments, we compared the JAK1 and 3D truncations with their respective overall structures. The results revealed that both the JAK1 and 3D truncations had minimal impact on their overall structures (Fig. S5A and B). In addition, we examined the effect of JAK1 truncations on other lysine residues and found that these truncations did not affect the accessibility of other lysine residues, which remained exposed on the surface (Fig. S5C).

To identify the critical amino acid residues governing the JAK1-3D interaction, we generated mutants of both JAK1 and 3D and performed Co-IP assays to assess their interaction. Wild-type JAK1 and mutants Y3A, D44A-R45A, N136A, T263A co-immunoprecipitated with 3D, while mutants R69A, T466A-S468A, and T470A-D471A showed reduced binding, and K205A displayed no interaction with 3D (Fig. 6B). This indicated that the K205 residue of JAK1 is a critical binding site for 3D. To rule out the possibility that the JAK1-K205A mutation might mask another potential binding site, we re-predicted the interaction between 3D and JAK1 with K205A substitution. Surprisingly, the binding energy between JAK1 and 3D was significantly reduced, and the binding position was altered. This further indicated that K205 is a critical site for the interaction between 3D and JAK1 (Fig. S5D). In addition, we compared the structure of the K205A mutant with the overall structure of JAK1. The results showed that the overall structure of JAK1 remained largely unchanged, and the previously predicted binding sites were still exposed on the surface. Therefore, the K205A mutation did not exert a masking effect on other potential binding sites (Fig. S5E).

Co-IP assays of 3D mutants revealed that wild-type 3D and mutants E7A, K87A, Q144A interact with JAK1, but the E76A, D90A, D148A, D150A mutants exhibited weakened interactions with JAK1 to varying degrees (Fig. 6C). Further investigation into the effect of 3D on the JAK1(K205A) mutant showed that 3D could not degrade this mutant, highlighting the importance of K205 in 3D-mediated JAK1 degradation (Fig. 6D). To determine whether mutation of K205 influences JAK1 ubiquitination regulated by 3D, we analyzed the impact of 3D on K48-linked ubiquitination of JAK1(K205A) and found that 3D failed to promote K48-linked ubiquitination of the JAK1(K205A) mutant (Fig. 6E). Collectively, these data suggest that the K205 residue of JAK1 plays a crucial role in promotion of JAK1 ubiquitination mediated by 3D, and residues E76, D90, D148, and D150 of 3D are important for the 3D-JAK1 interaction.

### Picornavirus 3D proteins broadly inhibited JAK-STAT signaling and promoted viral replication

**Fig 7:**
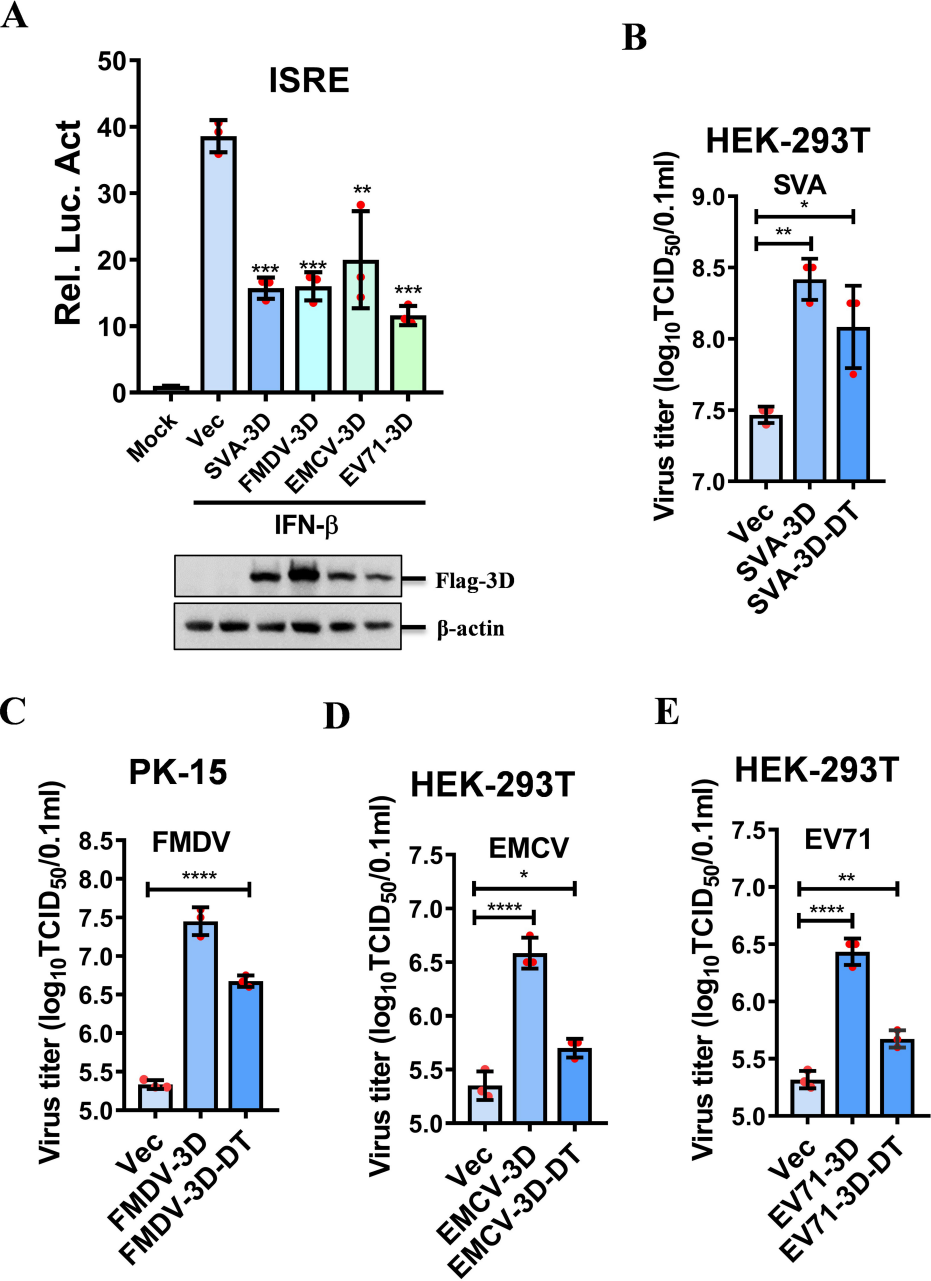
Picornavirus 3D proteins inhibited JAK-STAT signaling pathway and promoted viral replication**.** (**A**) HEK-293T cells were transfected with 50 ng of *ISRE* reporter plasmids, 5 ng of PRL-TK plasmids, and 50 ng of SVA-3D, FMDV-3D, EMCV-3D, or EV71-3D expressing plasmids for 24 h, followed by IFN-β treatment for 12 h. Luciferase activity was measured using the dual-luciferase assay system. (**B**) HEK-293T cells were transfected with empty vector, SVA-3D, or SVA-3D-DT expressing plasmids for 24 h, followed by infection with SVA (MOI = 0.5) for another 12 h. Cell culture supernatants were collected for determining viral titers. (**C**) PK-15 cells were transfected with empty vector, FMDV-3D, or FMDV-3D-DT expressing plasmids for 24 h, followed by infection with FMDV (MOI = 0.1) for 12 h. Cell culture supernatants were collected for determining viral titers. (**D, E**) HEK-293T cells were transfected with empty vector, EMCV-3D, EMCV-3D-DT (**D**), or EV71-3D, EV71-3D-DT (**E**) for 24 h, followed by infection with EMCV (MOI = 2) or EV71 (MOI = 2) for 24 h, respectively. Cell culture supernatants were collected for determining viral titers.

Given that SVA 3D inhibits JAK-STAT signaling, to investigate whether the 3D proteins from other picornaviruses share the ability to inhibit JAK-STAT signaling, we constructed expression plasmids for FMDV-3D, EMCV-3D, and EV71-3D and observed that all these 3D proteins could suppress IFN-β-induced *ISRE* promoter activity (Fig. 7A). Therefore, picornavirus 3D proteins broadly inhibited JAK-STAT signaling.

To explore whether the inhibition of the JAK-STAT signaling pathway by picornavirus 3D proteins translates into enhanced viral replication, we overexpressed SVA-3D protein in HEK-293T cells and observed that the presence of 3D protein upregulated SVA VP2 expression at the protein level (Fig. S6A), and the transcription of SVA increased as well (Fig. S6B). The replicating of SVA also enhanced in PK-15 cells (Fig. S6C and D). To further confirm that 3D promotes SVA replication by degrading JAK1 rather than playing the function of RdRp, we constructed a mutant plasmid SVA-3D-DT with enzyme active sites mutant of SVA-3D. The results showed that SVA-3D-DT had a weaker promoting effect on SVA than SVA-3D, and it still facilitated SVA replication (Fig. S6E). The infectious viral titration in the culture supernatants could also be detected, and SVA-3D could significantly upregulate SVA replication, while SVA-3D-DT had a slightly weaker effect on SVA replication (Fig. 7B). It suggested that SVA 3D protein not only facilitated viral replication by functioning as RdRp but also served as an antagonist of innate immunity, promoting viral replication.

To further investigate whether FMDV-3D, EMCV-3D, and EV71-3D share similar effects on viral replication. We constructed FMDV-3D, EMCV-3D, and EV71-3D enzyme active site mutants FMDV-3D-DT, EMCV-3D-DT, and EV71-3D-DT. We observed that both FMDV-3D and FMDV-3D-DT promoted FMDV replication, but FMDV-3D-DT had a weak effect on FMDV replication (Fig. S6F). The infectious viral titration in the culture supernatants shows a significant enhancement in FMDV replication in PK-15 cells by overexpression of FMDV-3D and FMDV-3D-DT (Fig. 7C). Overexpression of EMCV-3D and EV71-3D in HEK-293T cells revealed their ability to significantly promote viral replication, but EMCV-3D-DT and EV71-3D-DT only slightly improved viral replication (Fig. S6G and H). Meanwhile, the infectious culture supernatant was detected, which revealed that EMCV-3D and EV71-3D could significantly promote viral replication. However, the promoting effects of EMCV-3D-DT and EV71-3D-DT on their viral titers were relatively weak (Fig. 7D and E). Interestingly, SVA-3D-DT and FMDV-3D-DT showed a more potent effect on their respective viral replication compared to EMCV-3D-DT and EV71-3D-DT, suggesting that SVA-3D and FMDV-3D have stronger suppressive effects on the JAK-STAT pathway.

### The residues K205 and K249 in both human and porcine JAK1 played a significant role in suppression of SVA replication

**Fig 8:**
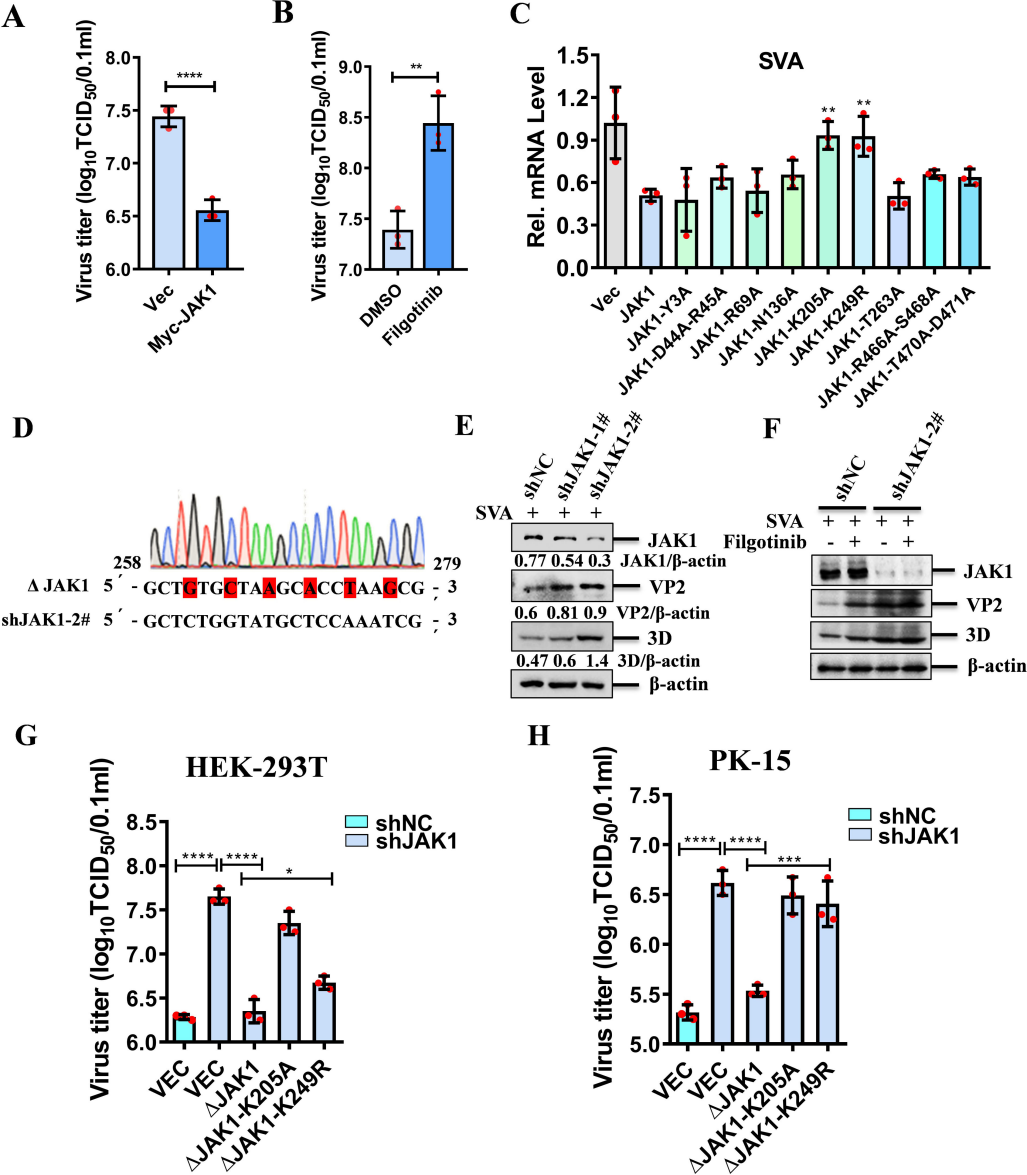
The impact of JAK1 residues K205 and K249 on SVA replication. (**A**) HEK-293T cells were transfected with empty vector or Myc-JAK1 for 24 h, followed by infection with SVA for 12 h. Cell culture supernatants were collected to determine the viral titers. (**B**) HEK-293T cells were treated with DMSO or the JAK1 inhibitor filgotinib for 6 h, followed by infection with SVA for 12 h. Viral titers were then determined. (**C**) HEK-293T cells were transfected with empty vector, JAK1, or JAK1 mutants (Y3A, D44A-R45A, R69A, N136A, K205A, K249R, T263A, T466A-S468A, T470A-D471A) for 24 h, followed by infection with SVA for 12 h. Total RNA was extracted, and SVA replication was quantified by qPCR analysis. (**D**) Schematic representation of constructing shRNA-resistant JAK1 mutant (ΔJAK1) by introducing a 6-nucleotide nonsense mutation in the target sequence of the shJAK1 plasmid. (**E**) The shRNA targeting JAK1 (shJAK1-1# and shJAK1-2#) or a non-targeting shRNA control (shNC) was synthesized and transfected into HEK-293T cells for 48 h. The knockdown efficiency was evaluated by western blotting. (**F**) HEK-293T cells were transfected with shNC or shRNA targeting JAK1 (shJAK1-2#) for 48 h, then treated with DMSO or filgotinib for 6 h, followed by infection with SVA for another 12 h. The cell lysates were analyzed by western blotting using the specified antibodies. (**G, H**) HEK-293T cells (**G**) or PK-15 cells (**H**) were transfected with shNC or shRNA targeting JAK1 (shJAK1-2#) for 48 h, followed by transfection with the shRNA-resistant JAK1 mutant (ΔJAK1, ΔJAK1-K205A, or ΔJAK1-K249R) expressing plasmids for another 24 h. The cells were then infected with SVA for 12 h. Cell culture supernatants were collected to determine the viral titers.

JAK1 plays a pivotal role in IFN response. To understand its role in SVA replication, we overexpressed JAK1 and observed that it suppressed the expression of SVA VP2 (Fig. S7A) and dose-dependently hindered the transcription of SVA (Fig. S7B). In addition, the viral titer of SVA was significantly reduced by overexpression of JAK1 (Fig. 8A). To further investigate the function of JAK1 in suppression of SVA replication, we utilized the JAK1 inhibitor filgotinib and found that SVA replication was enhanced at both the protein (Fig. S7C) and mRNA levels (Fig. S7D) in the presence of Filgotinib. The viral titer of SVA increased significantly when the JAK1 pathway was inhibited (Fig. 8B), indicating that the JAK-STAT signaling pathway is critical for inhibiting SVA replication.

Given preliminary findings on certain JAK1 residues, we explored their role in SVA replication by overexpressing mutants and found that the K205A and K249R mutations could abolish the inhibitory effect of JAK1 on SVA replication (Fig. 8C). The expression of JAK1 was verified by western blotting (Fig. S7E). Subsequently, we generated a series of mutants (ΔJAK1, ΔJAK1-R69A, ΔJAK1-K205A, ΔJAK1-K249R, ΔJAK1-R466A-S468A) with mutations at the targeted sequence of JAK1 shRNA to prevent degradation of these mutants in the JAK1 shRNA-transfected cells (Fig. 8D). Then, we utilized JAK1 shRNA to specifically knock down endogenous JAK1 expression. We observed that silencing JAK1 led to a significant upregulation of SVA VP2 and 3D protein levels. This suggests that JAK1 knockdown promotes SVA replication (Fig. 8E). JAK1 inhibitor filgotinib and JAK1 knockdown could promote SVA replication, we hypothesized that in JAK1-knockdown cells, filgotinib might further enhance SVA replication. To test this hypothesis, we investigated the effect of filgotinib on SVA replication in cells with JAK1 knockdown. Surprisingly, we found that filgotinib did not have a more pronounced promoting effect in this context. This lack of effect may be attributed to the already low levels of JAK1 expression in the knockdown cells, rendering filgotinib’s inhibitory action insufficient to exert a significant impact (Fig. 8F). In JAK1 shRNA-transfected cells, transfection of the constructed mutants revealed that ΔJAK1-K205A and ΔJAK1-K249R mutants had a reduced inhibitory effect on SVA replication compared to the ΔJAK1 (Fig. 8G), while ΔJAK1-R69A and ΔJAK1-R466A-S468A mutants exhibited antiviral effects comparable to ΔJAK1 (Fig. S7F and G). In addition, we aligned the sequences of human and porcine JAK1, which revealed the conservation of K205 and K249 (Fig. S8). Experiments with porcine JAK1 mutants in PK-15 cells mirrored the findings, showing no decrease in SVA viral titration in the cells overexpressing ΔJAK1-K205A and ΔJAK1-K249R mutants (Fig. 8H), and a similar trend in SVA VP2 and 3D expression was observed (Fig. S7H and I). These results indicate that residues K205 and K249 in both human and porcine JAK1 are crucial for their function in suppression of SVA replication.

## DISCUSSION

The innate immune system serves as the primary defense against viral invasions, activating the host antiviral responses. Upon viral infection, pattern recognition receptors (PRRs) detect viral components, triggering the production of IFNs, which stimulate the expression of ISGs. These ISGs directly exert antiviral effects, inhibiting viral replication (17). Throughout viral evolution, various strategies have been developed to evade or suppress host innate immunity. For example, the VP3 of FMDV degrades JAK1 throught the lysosome pathway and inhibits the activation of the type II IFN signaling pathway (10). Influenza PB2 protein inhibits the activation of the type I IFN signaling pathway by degrading JAK1 through the proteasome pathway (18). African swine fever virus (ASFV) MGF-505-7R promoted the degradation of JAK1 by upregulating the expression of E3 ubiquitin ligase RNF125 (19). In addition, ASFV MGF-360-10L enhanced the K48-linked ubiquitination of JAK1 by recruiting the E3 ubiquitin ligase HERC5 to degrade JAK1 and inhibit JAK-STAT pathway (20). SARS-CoV-2 causes cells to be less sensitive to IFN by targeting JAK1, TYK2, and IFNAR1 in the JAK-STAT pathway (21). Zika virus (ZIKV) NS2B-NS3 proteins target and degrade JAK1 through the proteasome pathway (22). Human cytomegalovirus (HCMV) inhibits IFN-α signaling pathways by inducing proteasome-dependent degradation of JAK1 (23).

Picornavirus 3D proteins function as RdRPs, facilitating viral replication without DNA intermediates. They also counteract host immune responses, induce autophagy, and activate inflammation to enhance viral replication. For instance, EV71 and coxsackievirus B3 3D proteins interact with MDA5, inhibiting IFN-β promoter activation and promoting viral replication (24). EV71 3D also degrades STAT1 to suppress IFN-γ signaling (25). Furthermore, the EV71 3D induces autophagy and promotes EV71 replication by interacting with Beclin1 (26). EMCV 3D is involved in inducing autophagy through the activation of the ER stress pathway and regulation of proteins associated with the UPR pathway (27). In terms of inflammatory responses, the EV71 3D can promote the assembly of the inflammasome complex, activating IL-1β by binding directly to NLRP3, forming a 3D-NLRP3-ASC complex (28). SVA 3D interacts with the NLRP3 NACHT domain, activating the inflammasome and inducing IL-1β secretion. In addition, the interaction between SVA 3D and IKKα, IKKβ leads to the activation of NF-κB and the promotion of IL-1β transcription (29). These actions demonstrate the diverse strategies by which picornavirus 3D proteins suppress host immune responses and promote their replication.

In this study, we observed that SVA 3D significantly inhibits the activation of IFN-β-induced *ISRE* promoter and IFN-γ-induced *GAS* promoter, and the expression of *ISGs* induced by IFN-β also decreased. Further investigation revealed that 3D induces K48-linked polyubiquitination of JAK1, leading to the degradation of JAK1, with the E3 ubiquitin ligase RNF125 playing a significant role in this process. SVA 3D has been reported to be ubiquitinated itself (30), to exclude the influence of 3D self-ubiquitination impacts interpretation of ubiquitination and luciferase assays. We generated the 3D-K169R-K321R mutant and assessed its effects on the activity of the IFN-β-induced *ISRE* promoter and on JAK1 ubiquitination. The results showed that the 3D-K169R-K321R mutant retained the ability to inhibit *ISRE* promoter activation. The mutant itself was not subject to ubiquitination, but it promoted the ubiquitination of JAK1.

**Fig 9:**
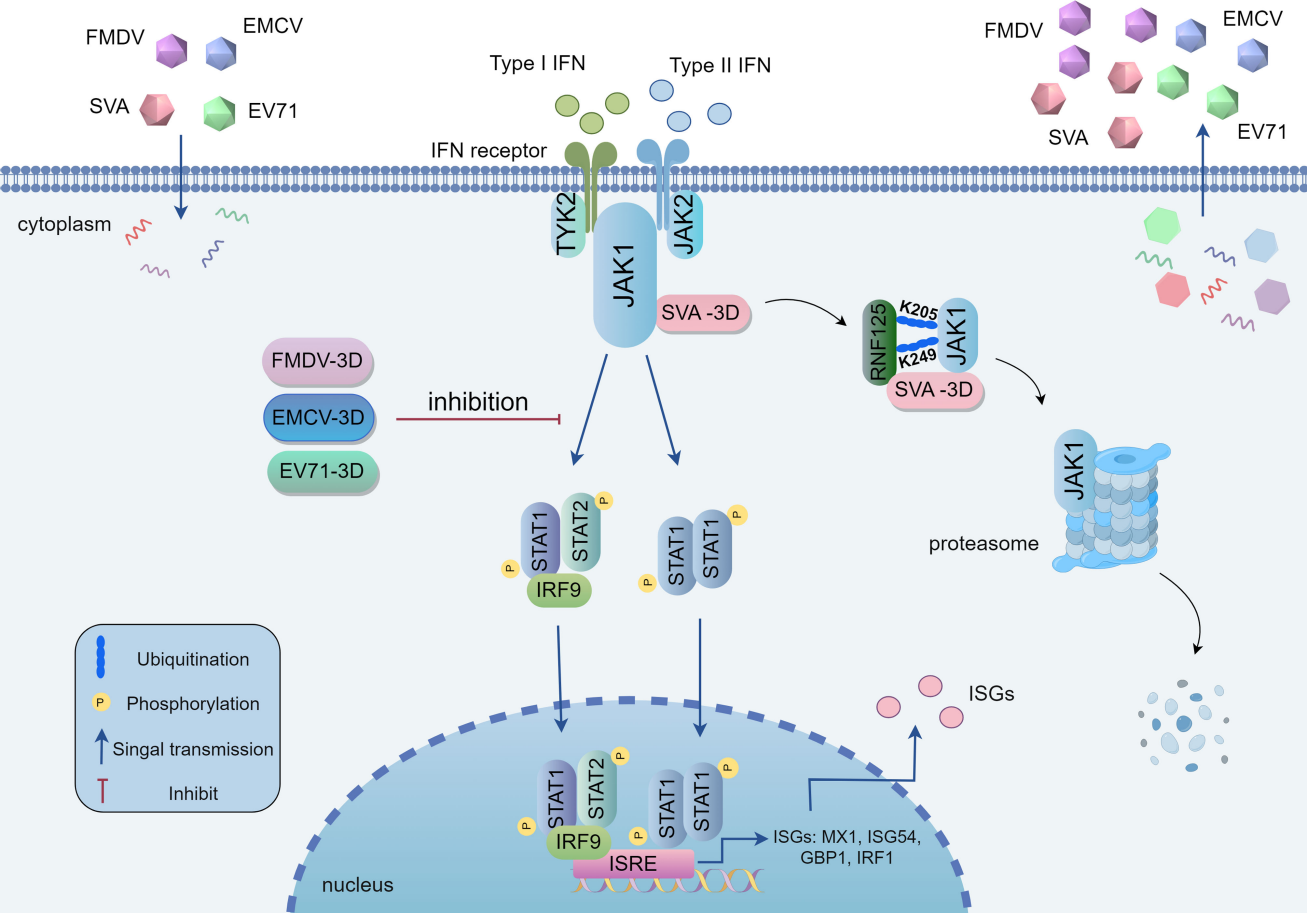
The mechanisms by which 3D proteins of picornaviruses target JAK1, impeding the activation of the JAK-STAT signaling cascade and facilitating viral replication. Upon SVA infection of host cells, the viral 3D protein interacts with JAK1 and recruits RNF125, promoting the K48-linked polyubiquitination of JAK1 at specific lysine residues (K205 and K249) and leading to its degradation. This results in the suppression of JAK-STAT signaling pathway activation, which counteracts the innate immune response and bolsters viral replication. EMCV-3D, EMCV-3D, and EV-71-3D also inhibit JAK-STAT signaling pathway activation for facilitating viral replication.

Further investigation showed that the JAK1 K205 residue and the 3D E76, D90, D148, and D150 residues are crucial for 3D-mediated degradation of JAK1. Specifically, the K205 and K249 residues of JAK1 are targeted by 3D for K48-linked ubiquitination. Moreover, we found that the K205 and K249 residues in both human and porcine JAK1 are critical sites for inhibiting SVA replication. In addition, we found that picornavirus 3D proteins broadly inhibit JAK-STAT signaling and promote viral replication. Notably, SVA-3D and FMDV-3D exhibited more potent effects on their respective viral replication compared to EMCV-3D and EV71-3D. In summary, we have uncovered a novel mechanism by which picornavirus 3D proteins target JAK1, antagonize the JAK-STAT signaling pathway, and enhance viral self-replication (Fig. 9).

## MATERIALS AND METHODS

### Cells, viruses, and infection

HEK-293T cells, PK-15 cells, HeLa cells, and BHK-21 cells were cultured in Dulbecco modified Eagle medium (DMEM; VivaCell) supplemented with 10% heat-inactivated fetal bovine serum (FBS; Excell) and maintained at 37°C (5% CO_2_). SVA CH-FJ-2017 strain (GenBank accession number KY747510), previously isolated and preserved in our lab, was utilized for viral infection (31). For virus propagation and titration, we employed IBRS-2 cells. The amplification and titration of FMDV type O strain O/BY/CHA/2010 were conducted using BHK-21 cells (32). The EV71 strain H (VR-1432) and EMCV strain, both previously stored in our laboratory, were propagated and titrated using HeLa cells. The 50% tissue culture infectious dose (TCID_50_) was determined using the Reed and Muench method (33). All virus strains were cryopreserved at −80°C. The viral infection experiments were conducted in accordance with the procedures described in previous studies (34).

### Plasmids and antibodies

The indicated SVA gene cDNA and the 3D gene cDNAs of FMDV, EMCV, and EV71 were individually cloned into the p3xFLAG-CMV-7.1 vector (Invitrogen) to generate plasmids expressing Flag-tagged viral proteins. Mammalian expression plasmids for Myc-tagged JAK1, JAK2, TYK2, STAT1, STAT2, and IRF9, and the IFN-β promoter luciferase reporter plasmids were kindly provided by Professor Hongbing Shu (Wuhan University, China). HA-tagged RNF125, STUB1, SOCS1, and SOCS3 were constructed using standard molecular biology protocols. A series of Flag-tagged truncated and site-specific mutants of 3D and Myc-tagged truncated and site-specific mutants of JAK1 plasmids were generated by site-directed mutagenesis PCR. All constructed plasmids were analyzed and verified by DNA sequencing.

The commercial antibodies used in this study include the following: anti-FLAG mouse Ab (Sigma), anti-Myc mouse Ab (Sigma), anti-HA mouse Ab (Invitrogen), anti-JAK1 rabbit Ab, anti-p-JAK1 rabbit Ab, anti-TYK2 rabbit Ab, anti-p-TYK2 rabbit Ab, anti-STAT1 rabbit Ab, anti-p-STAT1 rabbit Ab, anti-STAT2 rabbit Ab, anti-p-STAT2 rabbit Ab, anti-IRF9 rabbit Ab (Cell Signaling Technology), and anti-β-actin mouse Ab (Santa Cruz Biotechnology). Anti-3D rabbit polyclonal Ab and anti-VP2 rabbit polyclonal Ab were prepared by our laboratory previously (unpublished data). Goat anti-mouse or rabbit IgG (H + L) secondary antibodies were purchased from Biodragon Company.

### Transfection and reporter assays

HEK-293T cells, cultured in 48-well plates, were transfected with a combination of 50 ng of reporter plasmid, 5 ng of pRL-TK (Promega, Madison, WI, USA) serving as an internal control, and 50 ng of the specified plasmids. To standardize the transfection process, empty vector plasmids were included to ensure equal total plasmid amounts were introduced into each well. After 24 h post-transfection (hpt), the cells were stimulated with IFN-β (1,000 U/mL) or IFN-γ (1,000 U/mL) for a duration of 12 h. Following this treatment, whole-cell extracts were prepared to assess dual-luciferase activities. The experiment was conducted three times independently, with each trial executed in duplicate. The presented data reflect the mean values and standard deviations derived from these three independent experiments.

### RNA extraction and real-time PCR

Total RNA was extracted from the cell cultures using TRIzol Reagent (Vazyme), and then reverse-transcribed into cDNA with HiScript II Q RT SuperMix for qPCR (Vazyme) according to the manufacturer’s instructions. The relative amounts of cDNAs were quantified using ChamQ Universal SYBR qPCR Master Mix (Vazyme) following the manufacturer’s protocol to analyze the target genes. The glyceraldehyde-3-phosphate dehydrogenase (GAPDH) gene was used as an internal control, and the relative mRNA levels were calculated using the 2^−ΔΔCT^ method. The qPCR primers used in this study are shown in Table 2.

**TABLE 2:** The primers, siRNA, and shRNA sequences used in this study

Primers	Sequences (5′−3′)
SVA-3D	Forward: 5′-AGAATTTGGAAGCCATGCTCT-3′ Reverse: 5′-GAGCCAACATAGAAACAGATTGC-3′
Human JAK1	Forward: 5′-CTTTGCCCTGTATGACGAGAAC-3′ Reverse: 5′-ACCTCATCCGGTAGTGGAGC-3′
Human ISG54	Forward: 5′-ACGGTATGCTTGGAACGATTG-3′ Reverse: 5′-AACCCAGAGTGTGGCTGATG-3′
Human ISG56	Forward: 5′-TCACAGGTCAAGGATAGTC-3′ Reverse: 5′-CCACACTGTATTTGGTGTCTAGG-3′
Human MX1	Forward: 5′-TCTTCATGCTCCAGACGTAC-3′ Reverse: 5′-CCAGCTGTAGGTGTCCTTG-3′
Human OAS1	Forward: 5′-TCCACAGCCTCACTTCATTCC-3′ Reverse: 5′- ACATTAGACATTACCCTCCCATCAG-3′
Human CXCL9	Forward: 5′-CTGTTCCTGCATCAGCACCAAC-3′ Reverse: 5′-TGAACTCCATTCTTCAGTGTAGCA-3′
Human GBP1	Forward: 5′-CGAGGGTCTGGGAGATGTAG-3′ Reverse: 5′-T AGCCTGCTGGTTGATGGTT-3′
Human IRF1	Forward: 5′-GAGGAGGTGAAAGACCAGAGCA-3′ Reverse: 5′-TAGCATCTCGGCTGGACTTCGA-3′
Human STAT1	Forward: 5′- ATGGCAGTCTGGCGGCTGAATT-3′ Reverse: 5′-CCAAACCAGGCTGGCACAATTG-3′
Human GAPDH	Forward: 5′-CGGGAAGCTTGTGATCAATGG-3′ Reverse: 5′-GGCAGTGATGGCATGGACTG-3′
Porcine ISG15	Forward: 5′-GATCGGTGTGCCTGCCTTC-3′ Reverse: 5′-CGTTGCTGCGACCCTTGT-3′
Porcine ISG54	Forward: 5′-CTGGCAAAGAGCCCTAAGGA-3′ Reverse: 5′-CTCAGAGGGTCAATGGAATTCC-3′
Porcine OAS1	Forward: 5′-AAGCATCAGAAGCTTTGCATCTT-3′ Reverse: 5′-CAGGCCTGGGTTTCTTGAGTT-3′
Porcine MX1	Forward: 5′-CCTGTTGATGGTGCAAAGCT-3′ Reverse: 5′-TGCACATAGGCTTGAGGTCA-3′
Porcine JAK1	Forward: 5′-CATTATGCAAGGCGAGCACC-3′ Reverse: 5′-TCCTCAACACATTCGGGAGC-3′
Porcine GAPDH	Forward: 5′-ACATGGCCTCCAAGGAGTAAGA-3′ Reverse: 5′-GATCGAGTTGGGGCTGTGACT-3′
Human control-siRNA	Forward: 5′-UUCUCCGAACGUGUCACGU-3′ Reverse: 5′-ACGUGACACGUUCGGAGAA-3′
Human siRNF125 #1	Forward: 5′-GGAUCAUUGUAUUACUCAUTT-3′ Reverse: 5′-AUGAGUAAUACAAUGAUCCTT-3′
Human siRNF125 #2	Forward: 5′-GAAUGAAAUCAGAGUAUAATT-3′ Reverse: 5′-UUAUACUCUGAUUUCAUUCTT-3′
Human siRNF125 #3	Forward: 5′-CUGUAUUGCUACCAGUCUATT-3′ Reverse: 5′-UAGACUGGUAGCAAUACAGTT-3′
Human shJAK1#1	Forward:5′-GCCATCAATAAATTGCGGCAA-3′
Human shJAK1#2	Forward:5′-GCTCTGGTATGCTCCAAATCG-3′

### RNA interference (RNAi)

The siRNA and shRNA were transfected with jetPRIME DNA transfection reagent into HEK-293T cells. The RNF125 target sequence and shJAK1 were purchased from Sangon Biotech, China. An shRNA targeting green fluorescent protein was used as a negative control. The siRNA and shRNA sequences are shown in Table 2.

### Co-immunoprecipitation and western blotting analysis

HEK-293T cells were cultured in 10 cm dishes, and the monolayer cells were co-transfected with various plasmids. The collected cells were then lysed by radio-immunoprecipitation assay (RIPA) lysis buffer and immunoprecipitated with 50% (vol/vol) slurry of GammaBind G Plus-Sepharose (GE Health Care Life Sciences) binding with indicated primary antibodies (35). For western blotting analysis, target proteins were resolved by 10% SDS-PAGE and transferred onto the nitrocellulose membranes (Pall). The membrane was blocked by 5% nonfat-dried milk diluted in TBST and incubated with appropriate primary antibodies and secondary antibodies, and the antibody-antigen complexes were subsequently visualized using ECL detection reagents (Thermo Fisher Scientific).

### Confocal immunofluorescence assay

Cells were seeded into glass-bottom cell culture dishes and transfected with various plasmids using Lipofectamine 2000 according to the manufacturer’s instructions. At 24 hpt, the cells were infected with SVA for 10 h or stimulated with IFN-β (1,000 U/mL) for 30 min. The cells were then fixed, permeabilized, and blocked as previously described (36). The fixed cells were incubated with primary antibodies overnight, then incubated with secondary antibodies conjugated to Alexa Fluor 488 or Alexa Fluor 594 at room temperature for 2 h, cell nuclei were stained with 4′,6′-diamidino-2-phenylindole (DAPI) for 10 min. The cells were visualized using a Leica SP2 confocal microscopy system (Leica Microsystems, Wizla, Germany).

### Statistical analysis

All statistical analysis was performed using GraphPad Prism software version 8.0. A two-tailed Student’s *t* test and one-way ANOVA were employed to assess the significance of the data. The statistical significance is indicated in the figures (*P < 0.05, **P < 0.01, ***P < 0.001, ****P < 0.0001; ns indicates not significant).

## SUPPLEMENTAL MATERIAL

Supplemental figures mbio.01666-25-s0001.docx Figures S1 to S8.

ASM does not own the copyrights to Supplemental Material that may be linked to, or accessed through, an article. The authors have granted ASM a non-exclusive, world-wide license to publish the Supplemental Material files. Please contact the corresponding author directly for reuse.
