# Supplementary material for: Picornavirus 3D polymerase inhibits antiviral innate immunity by blocking the activation of JAK-STAT signaling pathway
Source: mBio. 2025 Sep 11;16(10):e01666-25. doi: 10.1128/mbio.01666-25 (PMC12506094; doi:10.1128/mbio.01666-25)
Supplement: Supplemental figures — Figures S1 to S8. [file mbio.01666-25-s0001.docx]

**Supplementary material**


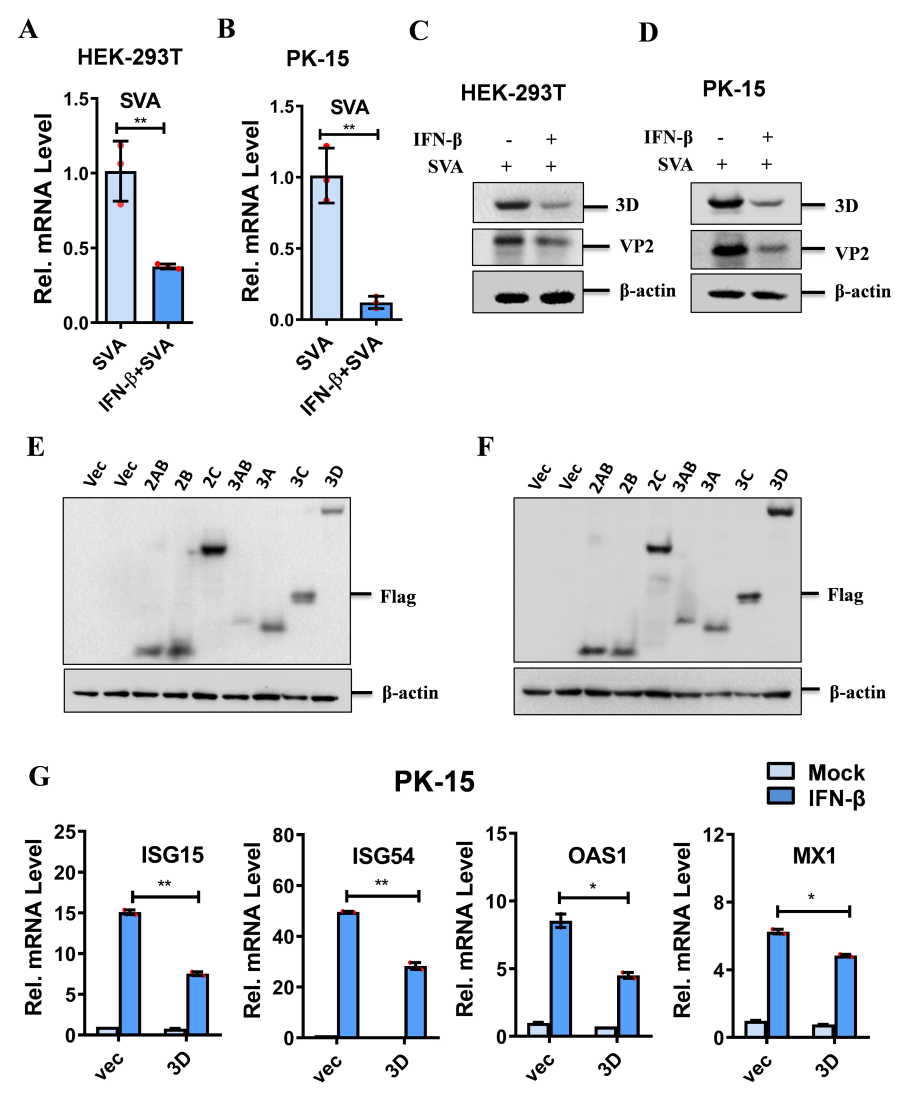


**Fig. S1 SVA 3D protein damaged JAK-STAT signaling pathway. (A-D)** HEK-293T cells or PK-15 cells were pretreated with human or porcine IFN-β (1000 U/mL) for 2 h, and the cells were then infected with GFP-SVA (MOI=0.5) for 12 h. The transcription of SVA 3D gene was detected by RT-PCR **(A, B)**; the protein expression levels of SVA 3D and VP2 were determined by western blotting **(C, D)**. **(E)** The expression of SVA viral proteins related to the main Figure 1D was verified by western blotting. **(F)** The expression of SVA viral proteins related to the main Figure 1E was verified by western blotting. (**G)** PK-15 cells were transfected with empty vector or Flag-3D expressing plasmids, followed by treatment with porcine IFN-β. The mRNA level of *ISG15*, *ISG54*, *OAS1* and *MX1* were detected by qPCR. All experiments were repeated for three times, yielding consistent outcomes. **P*<0.05, ***P*< 0.01, ****P*<0.001.


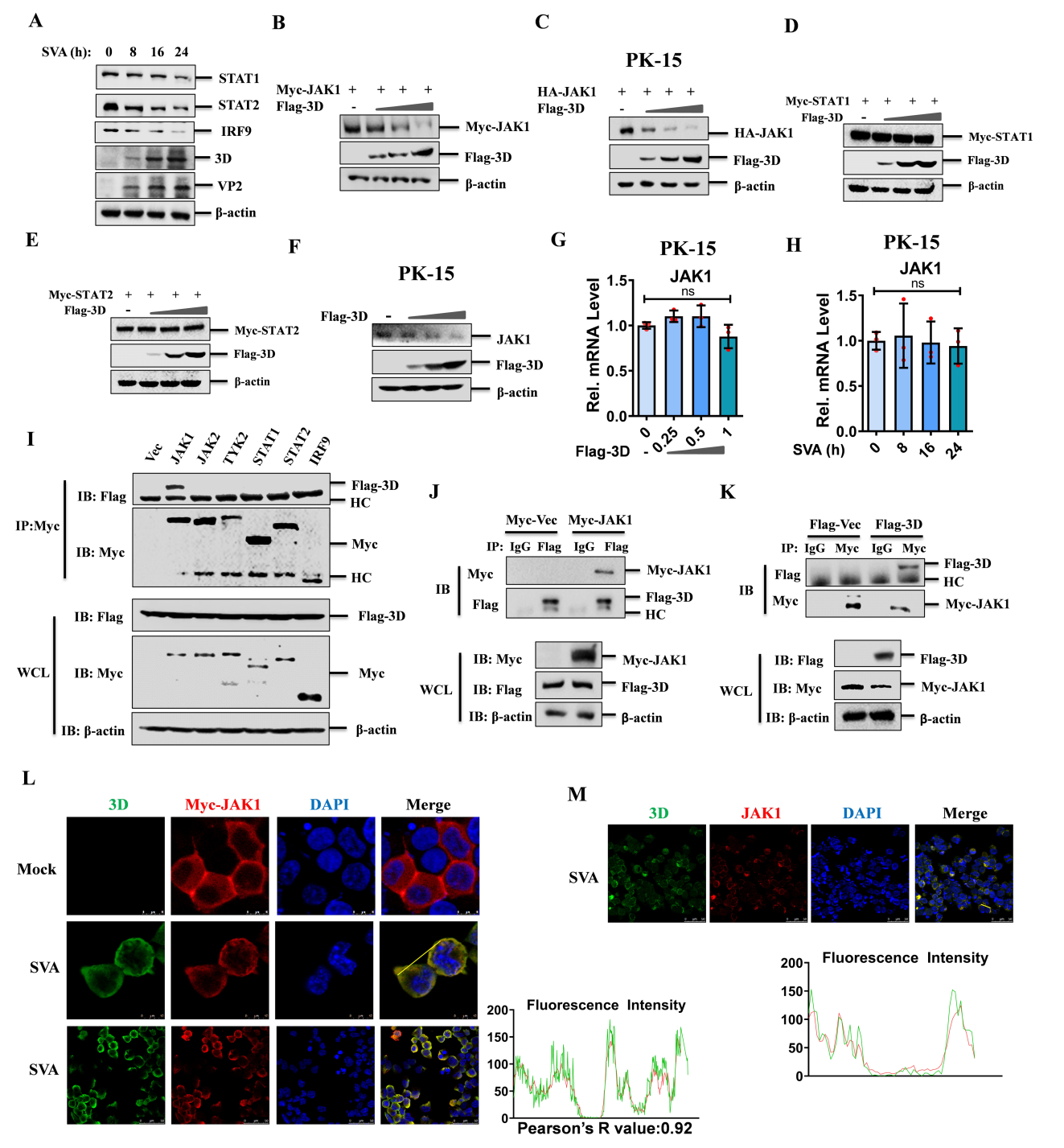


**Fig. S2 SVA 3D protein inhibited the expression of JAK1 and interacted with it. (A)** HEK-293T cells were infected with SVA (MOI=0.5) for 0, 8, 16 and 24 h respectively, and then analyzed by western blotting with the specified antibodies. **(B-C)** HEK-293T cells **(B)** or PK-15 cells **(C)** were transfected with 0, 0.25, 0.5 or 1 μg of Flag-3D expressing plasmids and 1μg of human Myc-JAK1 or porcine HA-JAK1 expressing plasmids for 24h, the cell lysates were analyzed by western blotting with the indicated antibodies. **(D-E)** HEK-293T cells were transfected with Flag-3D expressing plasmids (0, 0.25, 0.5 or 1 μg) and 1μg of Myc-STAT1 **(D)** or Myc-STAT2 **(E)** for 24 h. The cell lysates were analyzed by western blotting with the indicated antibodies. **(F, G)** PK-15 cells were transfected with 0, 0.25, 0.5 or 1 μg of Flag-3D expressing plasmids for 24 h. The protein expression levels of JAK1 were detected by western blotting **(F)**, the mRNA levels of JAK1 were analyzed by qPCR **(G).** **(H)** PK-15 cells were infected with SVA (MOI=1) for 0, 8, 16 and 24 h respectively, the mRNA expression levels of JAK1 were analyzed by qPCR. **(I)** The reverse Co-IP results from Figure 2G using the anti-Myc antibodies for immunoprecipitation. **(J, K)** HEK-293T cells were co-transfected with empty vector or Myc-JAK1 and vector or Flag-3D expressing plasmids for 36 h. The cell lysates were immunoprecipitated with anti-Flag **(J)**, anti-Myc **(K)** or control IgG antibodies, and the antigen-antibody complex was subjected to western blotting analysis. **(L)** HEK-293T cells were transfected with Myc-JAK1 for 12 h, followed by mock-infection or infection with SVA at an MOI of 0.1 for an additional 10 h. The subcellular localization of Myc-JAK1 and SVA-3D was evaluated by IFA. The nuclei were stained by DAPI (blue). Detected the fluorescence for 3D (green) and Myc-JAK1 (red). **(M)** The low magnification of Figure 2J.


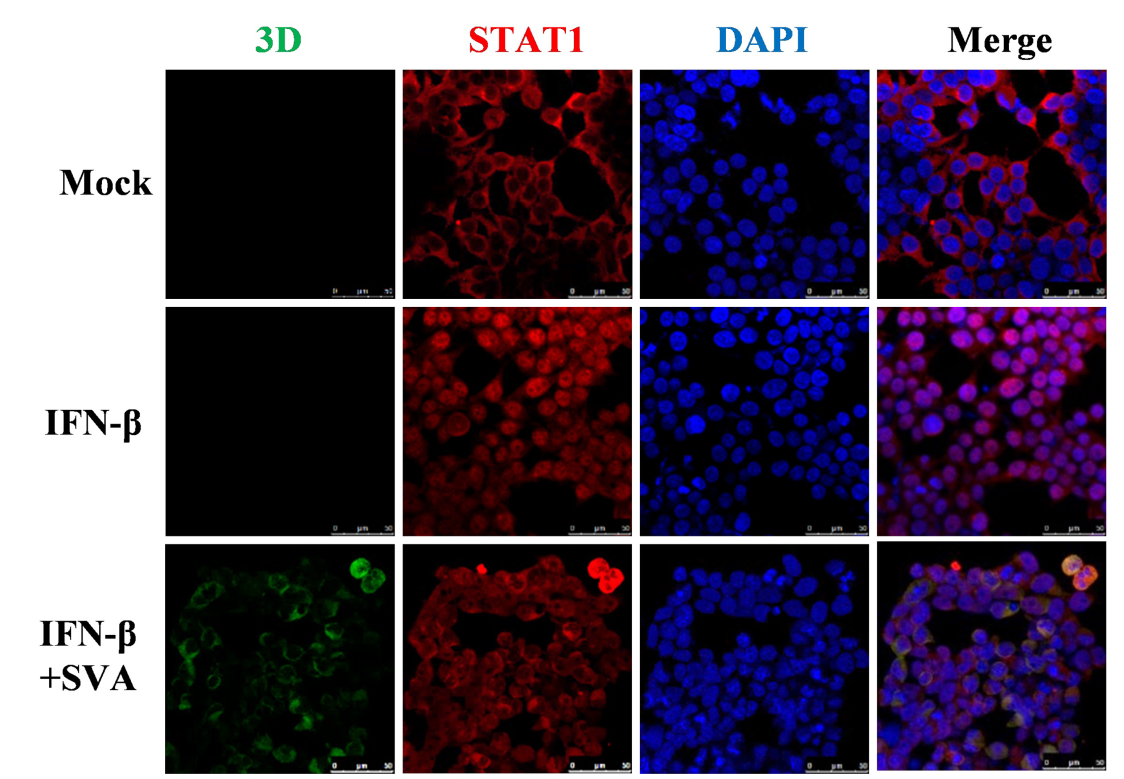


**Fig. S3 The low magnification of Figure 3E.**


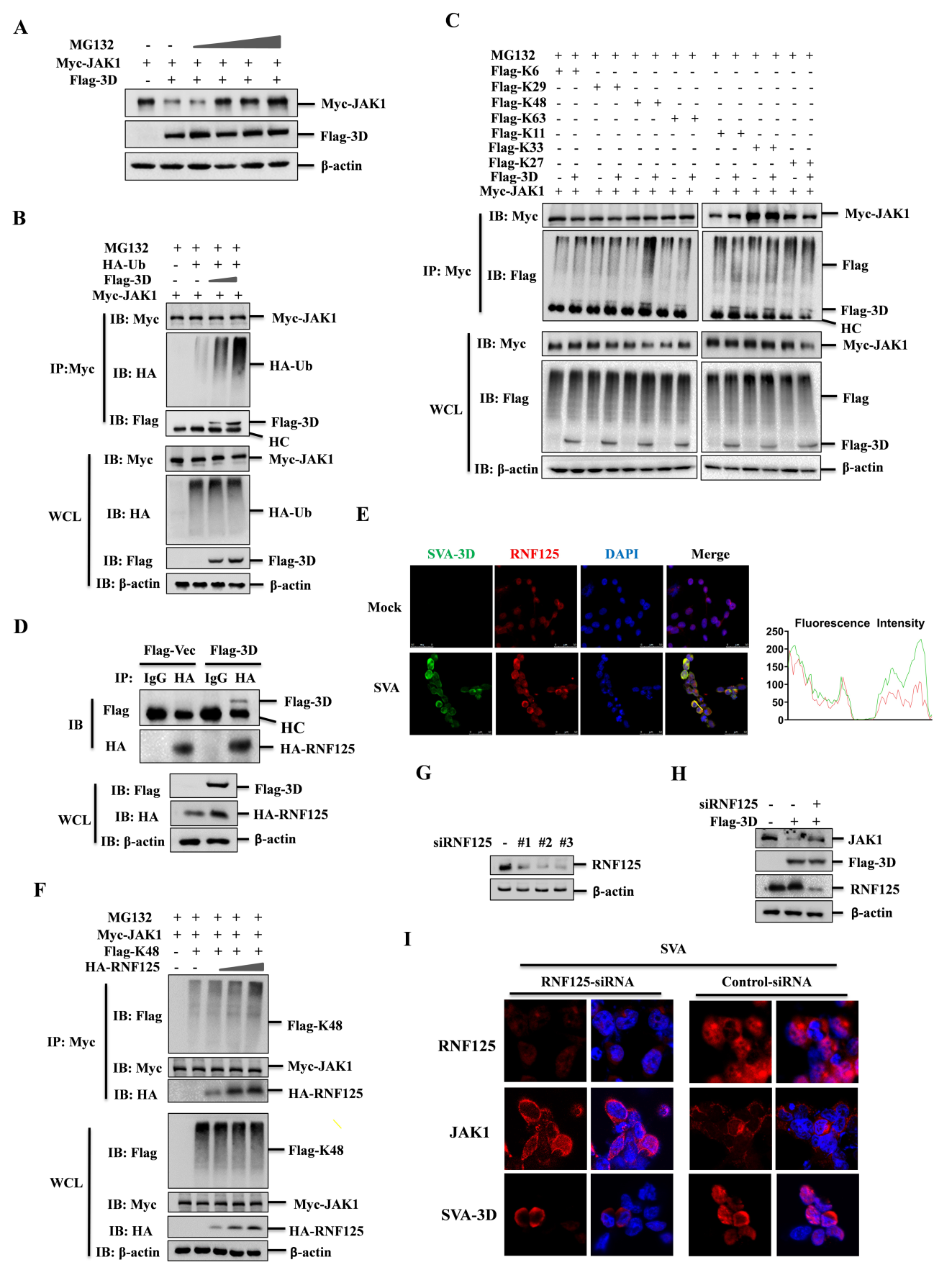


**Fig. S4 SVA 3D protein relied on RNF125 to degrade JAK1 via the proteasomal pathway. (A)** HEK-293T cells were transfected with empty vector or Flag-3D, and Myc-JAK1 expressing plasmids, and then maintained in the presence of escalating concentrations of MG132 (5, 10, 20, 50 μM). The cell lysates were analyzed by western blotting with the indicated antibodies. **(B)** The ubiquitination of JAK1 was analyzed by Co-IP assays. HEK-293T cells were co-transfected with Myc-JAK1, HA-ubiquitin (HA-Ub) or empty vector, and increasing amounts of Flag-3D expressing plasmids and treated with MG132. The cell lysates were immunoprecipitated with anti-Myc antibody. The immunoprecipitated proteins and WCL were subjected to western blotting analysis using the indicated antibodies. **(C)** The ubiquitination of JAK1 in HEK-293T cells was analyzed by transfecting the cells with Myc-JAK1, various Flag-ubiquitin mutants (K6-, K29-, K48-, K63-, K11-, K33-, or K27- only), and Flag-3D plasmids for 30 h, followed by a 6 h treatment with MG132. The immunoprecipitated proteins and WCL were subjected to western blotting analysis using the indicated antibodies. **(D)** The reverse Co-IP results from Figure 4D using the anti-HA antibodies for immunoprecipitation. **(E)** The low magnification of **Figure 4F. (F)** HEK-293T cells were transfected with Myc-JAK1, Flag-K48 or empty vector, and increasing amounts of HA-RNF125 expressing plasmids for 30 h, followed by MG132 treatment for 6 h. The interaction of Myc-JAK1, Flag-K48 and HA-RNF125 were analyzed by Co-IP assay. **(G)** The knockdown effects of three distinct RNF125 interfering RNAs were determined. **(H)** HEK-293T cells were transfected with RNF125 siRNA for 24 h, followed by transfection with the Flag-3D expressing plasmids for an additional 24 h. The expression levels of JAK1 were then measured by western blotting. **(I)** HEK-293T cells were transfected with Control siRNA or RNF125 siRNA for 36 h, and then infected with SVA (MOI=0.1) for 10 h. The expression of RNF125, JAK1 and 3D were dyed red in RNF125 knockdown and control cells and visualized through IFA respectively. Nuclei were stained with DAPI (blue).


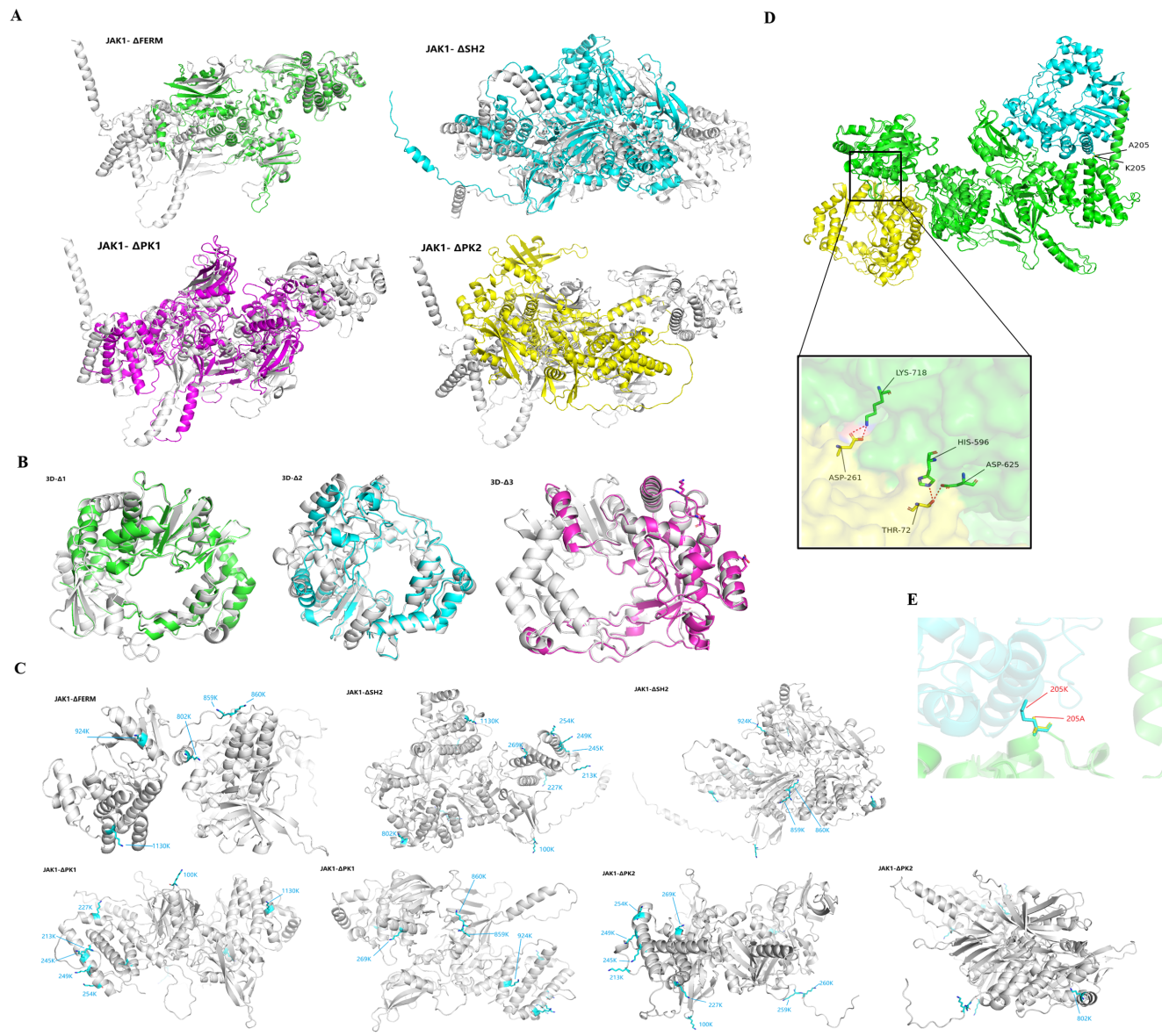


**Fig. S5 Predicting and verifying the interaction between JAK1 and 3D. (A)** The conformation of JAK1 truncations (JAK1-ΔFERM in green, JAK1-ΔSH2 in blue, JAK1-ΔPK1 in pink, JAK1-ΔPK2 in yellow) compared with the JAK1 overall structures. **(B)** The conformation of 3D truncations (3D-Δ1 in green, 3D-Δ2 in blue, 3D-Δ3 in pink) compared with the 3D overall structures. **(C)** The lysine residues of JAK1 truncations (JAK1-ΔFERM, JAK1-ΔSH2, JAK1-ΔPK1, JAK1-ΔPK2). **(D)** The interaction of JAK1-K205 and 3D(blue), and JAK1-A205 and 3D (yellow). **(E)** The conformation of JAK1-K205 and JAK1-A205.


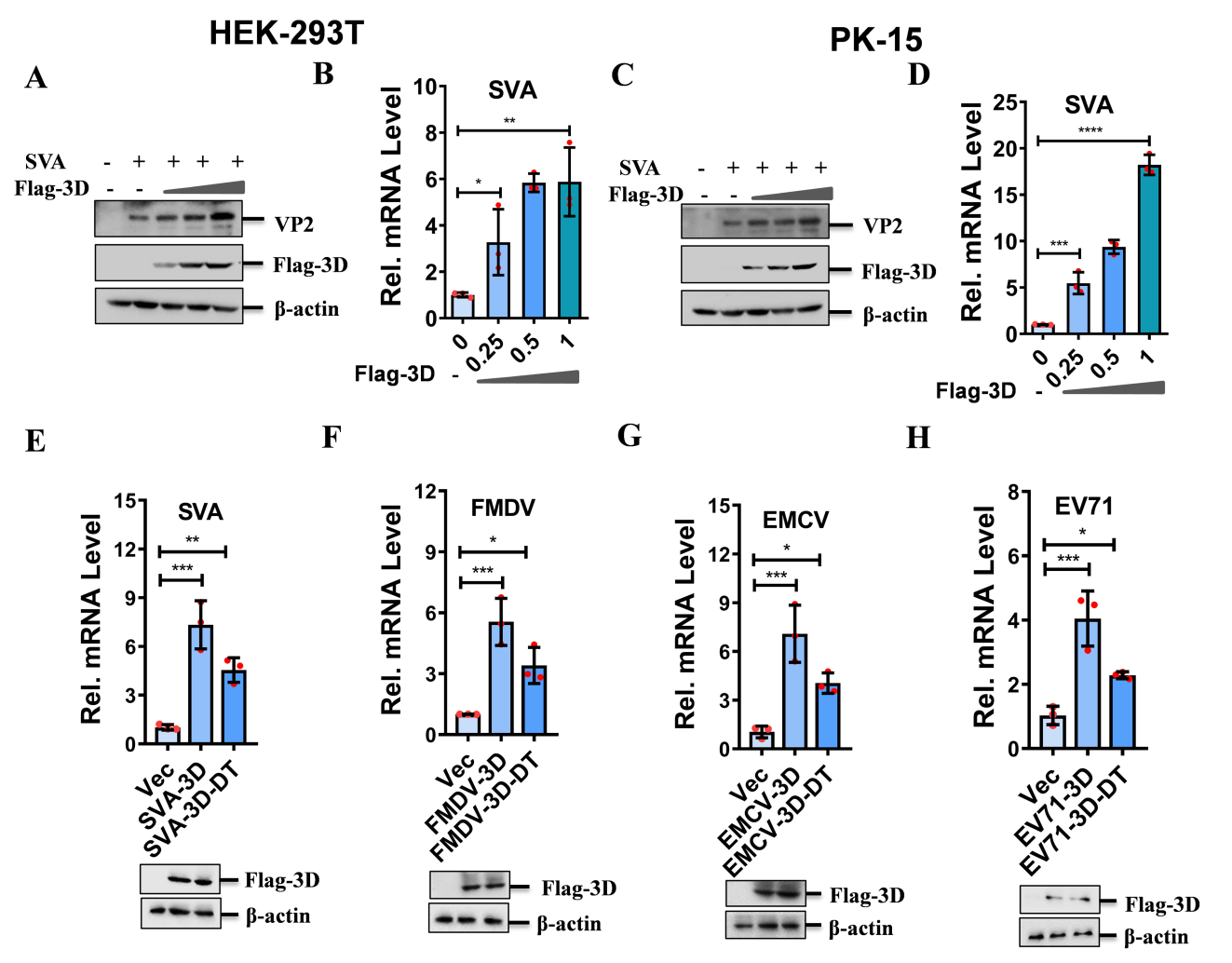


**Fig. S6 Picornavirus 3D proteins promoted viral replication. (A, B)** HEK-293T cells were transfected with increasing amounts of SVA Flag-3D expressing plasmids for 24 h, followed by SVA infection for an additional 12 h. The cells were lysed and analyzed by western blotting with the indicated antibodies **(A)**. Total RNA was extracted and analyzed by qPCR **(B)**. **(C, D)** Flag-3D expressing plasmids (0, 0.25, 0.5, or 1 μg) were transfected into PK-15 cells for 24 h, followed by SVA infection for another 12 h. The replication of SVA was analyzed by western blotting **(C)** and qPCR **(D)**. **(E)** HEK-293T cells were transfected with empty vector, SVA-3D, or SVA-3D-DT expressing plasmids for 24 h, then infected with SVA for 12 h. The expression of SVA mRNA was detected by qPCR. **(F)** PK-15 cells were transfected with empty vector, FMDV-3D, or FMDV-3D-DT expressing plasmids for 24 h, then infected with FMDV for 12 h. The expression of FMDV mRNA was detected by qPCR. **(G)** HEK-293T cells were transfected with empty vector, EMCV-3D, or EMCV-3D-DT expressing plasmids, followed by infection with EMCV, and the expression of EMCV mRNA was detected by qPCR. **(H)** HEK-293T cells were transfected with empty vector, EV71-3D, or EV71-3D-DT expressing plasmids, followed by infection with EV71, and the expression of EV71 mRNA was detected by qPCR.


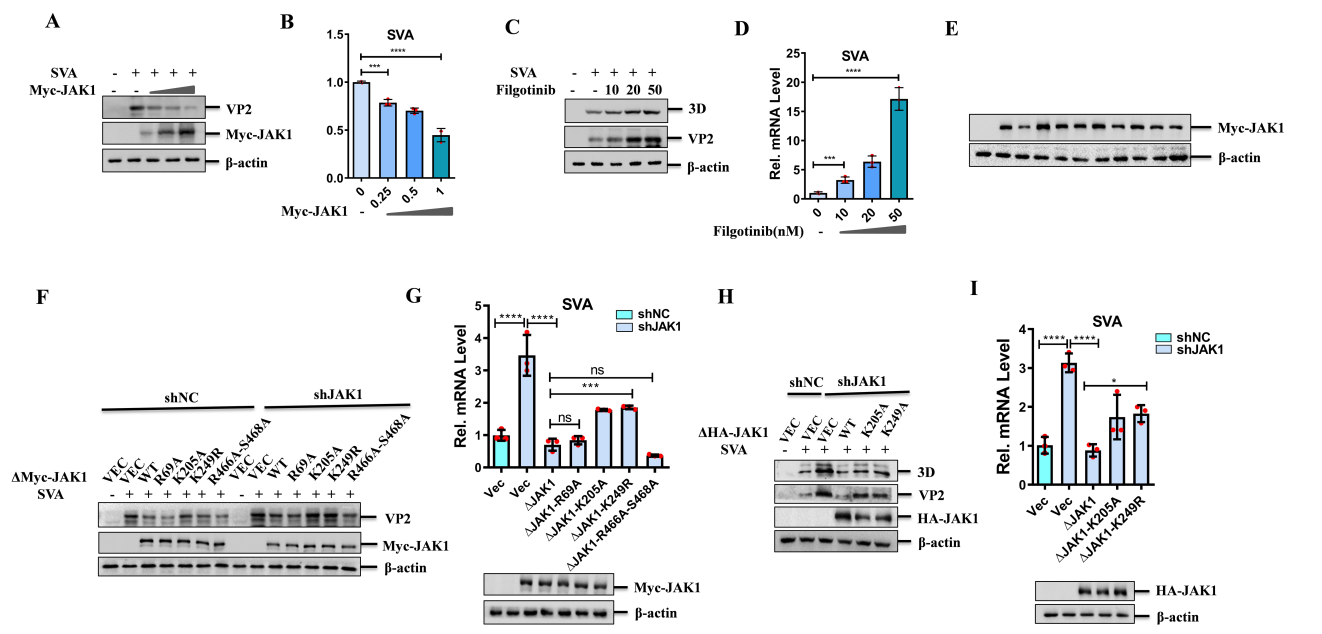


**Fig. S7 The residues K205 and K249 of JAK1 were critical for** **suppression of SVA replication. (A, B)** HEK-293T cells were transfected with increasing amounts (0, 0.25, 0.5, or 1 μg) of Myc-JAK1 for 24 h, and then infected with SVA for 12 h. The cells were lysed and analyzed by western blotting (**A**), and the replication of SVA at the mRNA level was analyzed by qPCR **(B)**. **(C, D)** Increasing amount of JAK1 inhibitor Filgotinib was used to treat HEK-293T cells for 6 h, followed by SVA infection for 12 h. The replication of SVA were detected at the protein level **(C)**, mRNA level **(D)**. **(E)** Expression of JAK1 mutants related to main Figure 8C. **(F, G)** HEK-293T cells were transfected with the shNC or shRNA of JAK1 for 48 h, followed by transfection with empty vector or shRNA off-target JAK1 mutants (ΔJAK1, ΔJAK1-R69A, ΔJAK1-K205A, ΔJAK1-K249R, ΔJAK1-R466A-S468A) for another 24 h, then infected with SVA for 12 h. The cells were lysed and analyzed by western blotting **(F)**, and total RNA was extracted and analyzed by qPCR **(G)**. **(H, I)** PK-15 cells were transfected with JAK1 shRNA or shNC for 48 h, followed by transfection with shRNA off-target porcine JAK1 mutants (ΔJAK1, ΔJAK1-K205A, ΔJAK1-K249R) for 24 h, and infected with SVA for 12 h. The cells were lysed and analyzed by western blotting **(H)**, and total RNA was extracted and analyzed by qPCR **(I)**.


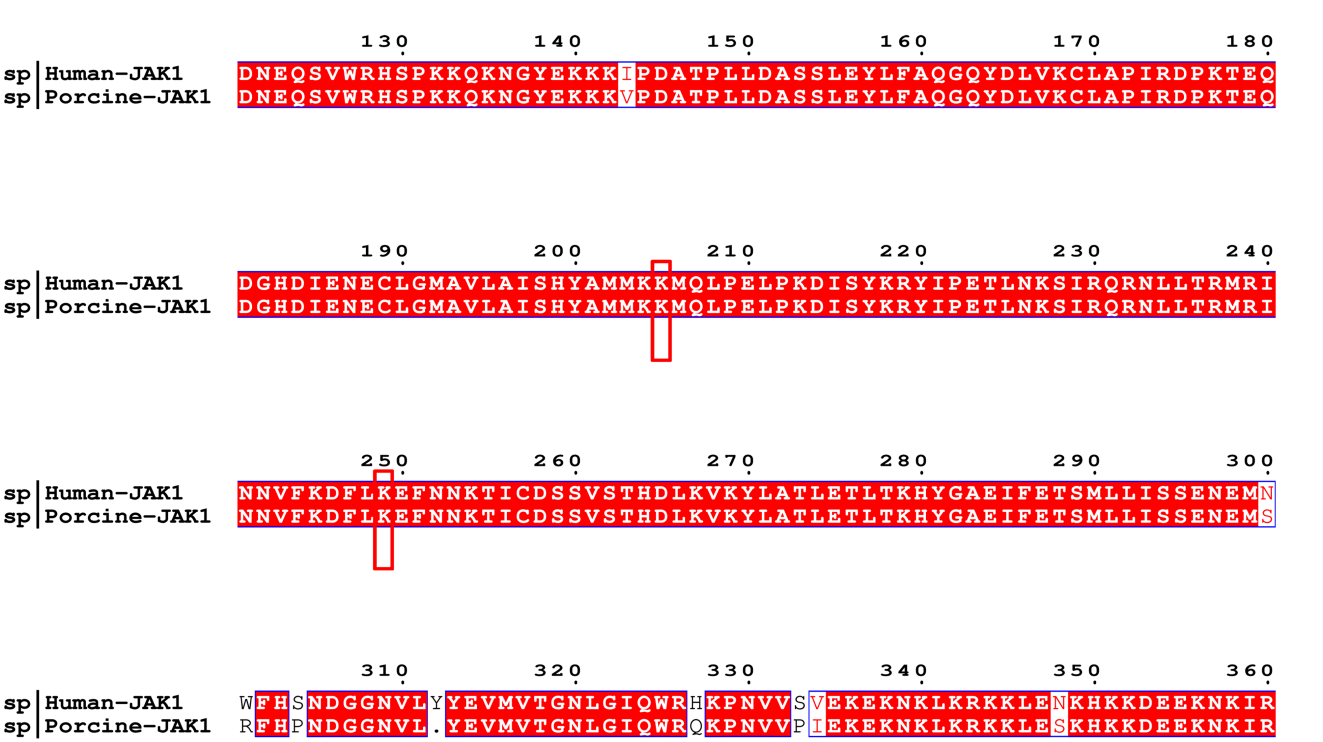


**Fig. S8 Sequence alignment of human and porcine JAK1 protein sequences.** The residues K205 and K249 were marked by red blocks. The strictly conserved residues are indicated by red blocks, and similar residues are enclosed in blue boxes.
